# Gene expression signatures of mutualism and pathogenesis in flax roots

**DOI:** 10.3389/fpls.2024.1415082

**Published:** 2024-10-10

**Authors:** Isadora Louise Alves da Costa Ribeiro Quintans, Eric Vukicevich, Vasilis Kokkoris, Erica Packard, Dinesh Adhikary, Miranda M. Hart, Michael K. Deyholos

**Affiliations:** ^1^ Departamento de Biociências, Universidade Federal Rural do Semi-Árido, Mossoró, Brazil; ^2^ Botany Department, Connecticut College, New London, CT, United States; ^3^ Amsterdam Institute for Life and Environment (A-LIFE), Faculty of Science, Section Systems Ecology, Vrije Universiteit Amsterdam, Amsterdam, Netherlands; ^4^ Department of Soil and Environment, Swedish University of Agricultural Sciences, Uppsala, Sweden; ^5^ Department of Agricultural, Food and Nutritional Science, University of Alberta, Edmonton, AB, Canada; ^6^ Irving K. Barber Faculty of Science, University of British Columbia, Kelowna, BC, Canada

**Keywords:** plant pathogen, arbuscular mycorrhizal fungi, bioprotection, plant defense, fusarium, transcriptomics

## Abstract

**Introduction:**

Fusarium wilt, a devastating soil-borne fungal disease in flax (*Linum usitatissimum*), is caused by *Fusarium oxysporum* f. sp. *lini*, a hemibiotrophic plant pathogen that penetrates plant roots. There are several reports of the molecular response of *L. usitatissimum* to *F. oxysporum* f. sp. *lini*; however, comparisons of the effects of mutualistic and pathogenic fungi on plants are more limited.

**Methods:**

In this study, we have integrated phenotyping and RNA-Seq approaches to examine the response of flax to *F. oxysporum* f.sp. lini and to a mutualistic arbuscular mycorrhizal fungus (AMF) *Rhizoglomus irregulare*. *R. irregulare* is a common soil fungus and also widely used as a commercial inoculant to improve plant growth. We measured flax growth parameters after plant inoculation with each or both fungi, in comparison with non-inoculated control. We performed transcriptome analysis of root tissues collected at 9 and 14 days post-inoculation.

**Results:**

We identified several differentially expressed genes (DEGs) in response to pathogenic and mutualistic fungi. These included genes related to ethylene and salicylic acid biosynthesis, carbohydrate binding, oxidoreductases, and sugar transmembrane transporters. Genes related to calcium signaling, nutrient transport, lipid metabolism, cell wall, and polysaccharide-modifying were up-regulated by *R. irregulare*; however, the same genes were down-regulated by *F. oxysporum* f. sp. *lini* when treated independently. In the combined treatment, genes related to cell wall modifications, hormone regulation and nutrient uptake were up-regulated. These results suggest that inoculation with *R. irregulare* reduced gene expression related to *F. oxysporum* f. sp. *lini* infection, leading to a reduced response to the pathogen. In response to AMF, flax prioritized mutualism-related gene expression over defense, reversing the growth inhibition caused by *F. oxysporum* f. sp.*lini* in the combined treatment.

**Discussion:**

This research provides insights into the protective effects of AMF, revealing the pre-symbiotic gene expression profile of flax in response to mutualism in comparison with pathogenicity. Potential target genes for crop improvement were identified, especially defense related genes.

## 1 Introduction

Fungi can have beneficial or harmful effects on plants. The mutualistic relationship yields several advantages for both partners, including nutrient exchange, plant disease protection, and a host environment that is conducive to fungal growth. Conversely, pathogenic fungi can cause significant damage by inhibiting plant growth and development, disrupting nutrient uptake, and leading to diseases that can greatly impact crop health. One of the major limitations to flax growth in North America is fusarium wilt, caused by the fungus *Fusarium oxysporum* f. sp. *lini* (Rashid, 2003). Currently, fusarium wilt is controlled by crop rotation, fungicides and resistant cultivars. Over 200 flax samples were recently evaluated for their susceptibility to wilt, showing a wide range of disease severity (Rozhmina et al., 2022). Most modern flax varieties have moderate to strong resistance to wilt, and genome regions associated with resistance have been mapped using genome-wide association studies, however, specific resistance genes have still not been identified (Kanapin et al., 2021).

Biological controls can help control phytopathogens and improve agricultural sustainability (Jacott et al., 2017; Jeffries et al., 2003). In this context the arbuscular mycorrhizal fungus (AMF) *Rhizoglomus irregulare*, which colonizes flax, represents a potential biocontrol agent, given its reported ability to suppress numerous phytopathogens (Akköprü and Demir, 2005; Ismail et al., 2013). AMF also increase the uptake of phosphate by plants, in parts by secreting organic acids and phosphatates (Lee et al., 2014; Majewska et al., 2017). However, the broader use of AMF to protect crops depends on several factors, including understanding the interaction between plants and mutualistic fungi (Ayaz et al., 2023). Beneficial fungi suppress pathogens in various ways including: secretion of enzymes that break down the structure of fungal cells (Lucini et al., 2019); competition for nutrients and space in the roots (Lucini et al., 2019); and stimulation of the plant immune system (Mukherjee et al., 2013). These mechanisms are not fully understood (Fiorilli et al., 2018). Pathogen suppression by *R. irregulare* may depend on priming the plant’s immune system, a phenomenon referred as ‘mycorrhiza-induced resistance’ (MIR) (Cameron et al., 2013; Conrath et al., 2015). Pathogen suppression may also involve hyperparasitism (Sorin et al., 2018).

Initial plant interactions with mutualistic fungi trigger plant immune responses, because plants may have difficulties distinguishing the initial recognition pattern of a mutualist from a pathogenic fungus (Zamioudis and Pieterse, 2012). However, AMF appear to have adapted to evade the plant’s immune system, and over time, a symbiotic relationship has developed (Zamioudis and Pieterse, 2012; Tisserant et al., 2014). By invaginating plasma membranes, AMF colonize root cortex cells forming arbuscules, which are sites for nutrition storage and exchange (Smith and Read, 2008). AMF expand the root absorption surface by developing an extraradical mycelium (Smith and Read, 2008), therefore improving phosphorus uptake, which is poorly mobile in the soil (Fiorilli et al., 2018; MacLean et al., 2017). By contrast, *Fusarium* species invade roots through wounds or natural openings, and penetrate plasma membranes, colonizing xylem vessels, blocking the vascular system and impairing water transport. The result is wilting, chlorosis, necrosis, browning of the vascular system, and ultimately, the death of the plant (Galindo-Gonzalez and Deyholos, 2016; Perez-Nadales et al., 2014). Understanding the molecular mechanisms of bioprotection will aid in the selection genes to improve plant resistance to pathogens.

Efforts to identify plant genes associated with symbiosis have advanced significantly with the availability of omics data, such as the genome assembly of *R. irregulare* (Tisserant et al., 2014). RNA-Seq has been used to investigate responses to AMF colonization, for example in: *Helianthus annuus* (sunflower) (Vangelisti et al., 2018), *Solanum lycopersicum* (tomato), *Lotus japonicus* (lotus) (Sugimura and Saito, 2017), *Zea mays* (maize) (Decouard et al., 2024), and *Triticum aestivum* (wheat) (Li et al., 2018) all inoculated with *R. irregulare*; and *Poncirus trifoliata* (hardy orange) inoculated with *Glomus versiforme* (An et al., 2018). These studies have identified genes associated with established mycorrhizal processes, such as membrane transport and cell wall modification (Vangelisti et al., 2018). A recent study analyzed the early interactions of *R. irregulare* and the non-host plant, *A. thaliana* (Fernandez et al., 2019). This study highlighted the significance of the signal exchange preceding the symbiotic relationship, leading to the expression of crucial symbiosis-related genes in non-host plants (Fernandez et al., 2019). These findings contribute to the understanding of plant-AMF interactions.

A previous RNA-Seq study of flax identified several defense gene transcripts that are more abundant in the varieties, Dakota and #3896, which are resistant to *F. oxysporum* f. sp. *lini* (Dmitriev et al., 2017). Prior to that (Galindo-Gonzalez and Deyholos, 2016), conducted RNA-Seq analysis of whole flax plants (variety CDC Bethune) inoculated with *F. oxysporum* f. sp. *lini* and identified 204 differentially expressed genes (DEGs) at 8 days post-infection (DPI), and 1,043 DEGs at 18 DPI with *F. oxysporum* f. sp. *lini*. CDC Bethune has moderate resistance to *F. oxysporum* f. sp. *lini* (Rowland et al., 2002; Galindo-Gonzalez and Deyholos, 2016). These DEGs were associated with hormone production, flavonoid production, lignin production, ROS production, transcription factors, and ethylene response factors. These findings complement the objectives of our current study by providing insights into flax responses to fungal pathogen infection. Our study focuses on identifying gene expression signatures in flax roots in the presence of a phytopathogenic fungus (*F. oxysporum* f.sp. *lini*) in combination with an arbuscular mycorrhizal mutualist (*R. irregulare*). This is the first transcriptomic investigation on the tripartite interaction between *L. usitatissimum*, *F. oxysporum* f. sp. lini, and *R. irregulare*. By investigating gene expression profiles and the regulatory networks of these plant-fungal interactions, we can reduce the gap between known bioprotection mechanisms and the genes that regulate them. Furthermore, many previous studies have overlooked the pre-symbiotic stages of exposure to pathogenic fungi, and the combined effects of *R. irregulare* and *F. oxysporum* f. sp. *lini* on flax. We hypothesize that biologically significant genes will exhibit different expression patterns when flax is exposed to a pathogenic fungus (*F. oxysporum* f. sp. *lini*), compared to a mutualistic fungus (*R. irregulare*), and when these fungi are combined. We further assume that pre-symbiotic interactions, which are the initial interactions between plant and fungi before symbiosis is established, are crucial for understanding the trajectory of a mutualistic or pathogenic relationship between fungi and plants. To test our hypothesis, we analyzed the bioprotective effects of *R. irregulare* upon *F. oxysporum* f. sp. *lini* in flax in an in-depth, multi-level study, since these effects remain heretofore unexplored. The DEGs identified here provide further insight on the molecular differences between mutualism and disease outcomes.

## 2 Materials and methods

Transcriptome responses of flax roots to two fungi were analyzed. We performed four different combinations of inoculations: i) control (mock-inoculated); ii) *R. irregulare*; iii) *F. oxysporum* f. sp. *lini*; and iv) the combination of *R. irregulare* and *F. oxysporum* f. sp. *lini*.

First, glass tubes (25 x 200 mm) were prepared for all treatments. The tubes were filled with 3 g of vermiculite mixed with 33 mg of *R. irregulare* inoculum (autoclaved or not) and 10 mL of 10% of Murashige-Skoog liquid medium (MS basal medium Sigma–Aldrich, St. Louis, MO, USA), in sterile conditions. To control for any effects of non-AMF components in the inoculum, in the non-AMF treatments, the same mass of inoculum was added following autoclaving of the inoculum (121°C for 90 minutes, in two successive cycles). Flax seeds were sown in the test tubes and immediately 1 mL of *F. oxysporum* f. sp. *lini* spore suspension was added to the soil surface (alone and combined treatments). In non-pathogenic assays (control, and AMF alone), 1 mL of sterile water was added to the soil surface. Tubes were placed in a growth chamber at 22°C with a 16 h day/8 h night photoperiod. Harvest was performed at 9 and 14 days post inoculation (dpi), and 12-20 plants were harvested per treatment/time point, depending on the germination rates.

### 2.1 Fungal material

#### 2.1.1 Pathogenic fungi

*Fusarium oxysporum* f. sp. *lini* (isolate 81) (Galindo-Gonzalez and Deyholos, 2016) was provided Dr. Khalid Rashid (Agriculture and Agri-Food Canada, Morden, MB, Canada). This isolate was described as aggressive in a previous study (Galindo-Gonzalez and Deyholos, 2016). *F. oxysporum* f. sp. *lini* isolate was grown for two weeks on PDA medium in the dark, at room temperature. The spores were collected after flooding each plate with 0.5% (v/v) Tween 20 (Sigma–Aldrich, St. Louis, MO, USA) in sterile water. A hemocytometer was used to count the spores. The spores were diluted with 0.5% (v/v) Tween 20 in sterile water to the concentration of 10^5^ spores mL^−1^ for flax inoculation.

#### 2.1.2 Beneficial fungi

The isolate of *Rhizoglomus irregulare* DAOM197198 was obtained as a commercial inoculum (AGTIV^®^ Specialty Crops powder, Premier Tech, Rivière-du-Loup, Canada). To each test tube, we applied 33 mg of the powder containing the spores, which corresponded to approximately 396 viable spores (12 000 viable spores/g), according to the manufacturer. As previously described, 33 mg of the autoclaved powder was used in non-AMF inoculations.

### 2.2 Plant material

Seeds of *Linum usitatissimum* var. CDC Bethune were grown according to Galindo-Gonzalez and Deyholos (2016). We chose this variety because it is widely cultivated in Canada, shows moderate resistance to *F. oxysporum* f. sp. *lini*, has been used previously in our laboratory for plant-fungal interaction studies, and is the source of the flax reference genome (Wang et al., 2012; Galindo-Gonzalez and Deyholos, 2016; You et al., 2016). Flax seeds were surface disinfected with 70% ethanol for one minute, immersed in 9.6% bleach for five minutes, and rinsed six times in sterile distilled water for one minute each. The seeds were dried in a laminar flow hood after rinsing and sowing.

### 2.3 Disease symptoms analysis and plant growth assessment

At 9 and 14 dpi, 12-20 plants from each treatment were harvested for further experiments. These sampling times were chosen because the plant and pathogen appeared to be interacting at 9 dpi, without yet causing severe wilt by 14 dpi, based on preliminary data and previous experiments (Galindo-Gonzalez and Deyholos, 2016). After harvest, the root system was gently washed to remove any substrate and the total fresh weight and shoot length for each plant were measured. Roots were then separated from the shoots. Three root systems from each inoculated plant were kept in 15 mL conical polypropylene tubes with ethanol (35% v/v) until further morphological analysis. The remaining roots were flash frozen in liquid nitrogen and kept in 2 mL tubes for RNA-Seq analysis (See section 2.6.1).

The morphological analysis of the roots was performed with the aid of a large-format scanner (Epson expression 11000XL) and the digital images were processed with the program WinRHIZO (Arsenault et al., 1995). The parameters root length, branching, or necrosis were analyzed. Color analysis was performed to assess the degree of root necrosis caused by *F. oxysporum* f. sp. *lini*. The color classes were scored according to the method described in Kokkoris and Hart (2019).

### 2.4 Statistical analysis of growth parameters

Morphological parameters were measured at harvest and prior to flash freezing and RNA extraction for the sample individuals. Since we were interested in how the presence of each fungus or combination of fungi differed from each other in plant responses, relative to a mock inoculation, we first normalized each inoculated plant’s phenotypic growth response to the mean of the control group for each parameter. Each measurement/data point was normalized to the uninoculated control growth/phenotype by subtracting the mean of the control group for each growth response (i.e. shoot growth, root growth, biomass, etc.) We then compared these relative growth parameters among fungal treatments.

Differences among treatments in shoot length and total biomass were assessed using a multivariate analysis of variance (MANOVA) in the ‘R’ base package (R statistical computing) (R Development Core Team, 2013). *Post-hoc* univariate analyses of variance (ANOVAs) and Tukey’s honest significant difference (Tukey, 1949) were then used to assess which growth parameters differed among the treatments. Because only a subset of samples were processed for root traits, a separate MANOVA was used to determine if treatment had any effect on root traits including total root length, branching intensity (measured as number of tips per cm of root length), or root necrotic surface area. Due to the low sample size for these root analyses, MANOVA was assessed at an α of 0.10. *Post-hoc* tests for treatment effects on root traits were as above. Samples from different harvest times (9 and 14 dpi) were analyzed separately to allow for association of growth responses with gene expression data on each date.

### 2.5 Root colonization assessment

#### 2.5.1 Fungal isolation

To confirm that the symptoms observed were caused by fungal infection, *F. oxysporum* f. sp. *lini* was re-isolated from the roots of the inoculated plants. Root sections from the control and each treatment were surface sterilized in 0.5% sodium hypochlorite for 30 seconds and then immediately washed with sterile distilled water for 30 seconds. Six to seven root sections were transferred to PDA medium and grown for 7 days in the dark at room temperature. Subsequently, plates were examined for fungal growth. We verified that the colonies and spore morphology were consistent with the original inocula, using microscopy.

#### 2.5.2 Microscopy

Flax roots inoculated with *R. irregulare* were stained to evaluate the extent of colonization according to the method of Vangelisti et al. (2018). Roots were washed, cleared in 10% KOH for 5 min at 90 °C and then rinsed three times with Reverse osmosis (RO) water. Cleared roots were stained for 3.5 min in ink-vinegar (5%) Sheaffer ink at 90 °C. Roots were destained for 25 min in RO water with few drops of vinegar. These roots were then mounted in microscope slides to visualize the fungal structures inside the roots.

### 2.6 Transcriptome analysis of flax roots inoculated with symbiotic fungi

Transcriptome responses were assessed for flax roots inoculated with either *F. oxysporum* f. sp. *lini* (isolate 81) (Galindo-Gonzalez and Deyholos, 2016)*, R. irregulare*, *R. irregulare* combined with *F. oxysporum* f. sp. *lini*, or control mock-inoculated plants. Harvest was performed as described above, and 6-7 root samples were pooled per treatment group, resulting in three biological replicates for each treatment. Disease symptoms and growth measurements were assessed, and roots were excised and immediately frozen in liquid nitrogen. Total RNA was extracted from pooled samples.

#### 2.6.1 RNA extraction and cDNA synthesis

The frozen tissues were lysed in liquid nitrogen using the Tissuelyser II instrument (Qiagen, Valencia, CA, USA). The RNA for each pooled sample was isolated using E.Z.N.A.^®^ Total RNA Kit I (Omega Bio-tek, Inc.). The enzyme RNase-free DNase Set I (Omega Bio-tek, Inc.) was used to remove DNA contamination and the RNA quality was evaluated using a NanoDrop 1000 Spectrophotometer (Thermo Fisher). The absence of contaminating genomic DNA was verified in a 1% agarose gel. Subsequently, double-stranded cDNA was synthesized from 1 μg of the total RNA with the qScript cDNA Synthesis Kit (Qiagen, Beverly Inc, MA, USA).

#### 2.6.2 RNA sequencing

Sequencing was performed for each treatment/time point combination, in three replicates, for a total of 24 independent RNA sequencing reactions by the service provider, BGI (Shenzen, China). Approximately 5 μg of total RNA of each sample was sent to BGI where the quality and quantity of RNA were evaluated using an Agilent 2100 Bioanalyzer. Library preparation was performed using mRNA enrichment (using oligo (dT) magnetic beads), and the target RNA was obtained after purification, fragmented and reverse transcribed to double-strand cDNA (dscDNA) with a N6 random primer. End repair and A-tailing were performed on the dscDNA to ligate the sequencing adapters to the fragments. The ligation product was amplified using two specific primers and subjected to the following single-strand circularization process. The PCR fragments products were heat-denatured and the single strand DNA was cyclized by splint oligo (which is a molecule reverse-complemented to one special strand of the PCR product) and the single-strand molecule was ligated using DNA ligase forming the DNA nanoballs (DNB). Single end sequencing was performed using the BGI-SEQ 500 instrument.

### 2.7 Bioinformatic analysis

Fastq data files were mapped to the flax genome (Wang et al., 2012) using TopHat v2.1.0 (Kim et al., 2013). The mapped sequence reads were used as inputs to cufflinks (Trapnell et al., 2010), to assemble the transcriptome from the RNA-Seq data and quantify their expression. Cufflinks was run with the GTF-guide option (reference transcript annotation to guide assembly), using a file with previously annotated genes. The gtf files of the transcripts of all treatments and replicates were combined using the cuffmerge program (Trapnell et al., 2010). The cuffdiff program (Trapnell et al., 2010) was used to compare the control (uninoculated) treatment with flax plants inoculated with the individual fungal isolates or their combination at 9 and 14 dpi. Transcript abundance was calculated as FPKM (fragments per kilobase of transcript per million mapped reads). Differential expression was calculated as the log2 fold-change ratio between the treated and control FPKMs, and ratios with FDR (q<0.05) were defined as differentially expressed genes (DEGs). Gene Ontology (GO) enrichment was calculated in AgriGO (Tian et al., 2017) using default parameters.

### 2.8 Quantitative real time PCR validation

To validate the results from the RNA-Seq, quantitative real time PCR (qRT-PCR) primers were designed for genes that responded significantly (FDR<0.05, FC 2) to *F. oxysporum* f. sp. *lini* inoculation. The qRT-PCR reactions were then performed, including all the treatments and timepoints, using the Perfecta Mix kit (QIAGEN, Beverly Inc, MA, USA) following the manufacturer’s recommendations. The ubiquitin gene was used as a reference gene, as described by Galindo-Gonzalez and Deyholos (2016). Prior to performing qRT-PCR, the amplification was optimized by gradient PCR and the amplicons of the PCRs were visualized on 1% agarose gels. Using cDNA (1: 6 dilution, 1.0 μl) as template. Sequences and annealing temperatures of the primers used are reported in **Table 1**.

**Table 1:** Primers used for qRT-PCR analysis.

Gene_ID	Forward	Reverse	Temperature
–	CCA AGA TCC AGG ACA AGG AA	GAA CCA GGT GGA GAG TCG AT	54°C
Lus10002741.g	TGT TAT GGG TGG TGG TAG T	CTT GCA AGC TCG TAA CCC	54°C
Lus10004410.g	ACT GTC CCT TCA CGG TAT	AGG ATC GCT GGG AAA GTA	54°C
Lus10005358.g	ACT ACA CCC TCC CGA TAA G	CCA CGA CAG CAT GAG AAA T	54°C
Lus10005858.g	CA CGG AGG ACG ATC TTT	TAT TTC CGC CGC TTC ATC	54°C
Lus10006691.g	TAG TGG CCA AGT GCA AAG	GGG AAG GCC TCA ACA ATA AT	54°C
Lus10009254.g	AAT CGC CGG ATT CAA CAG	CGC CTT CGT CAG AAC ATT AT	54°C
Lus10010696.g	GAC GGT TGT ATG TGG GAT AG	GAG CAA CGG AGC CTT ATT	54°C
Lus10012880.g	CTC CCG CTA AAC CAA TCA A	CGG AGA CGT AAG CCA AAT AA	54°C
Lus10014241.g	CGG CCA CAG CTG TTT AT	GAA GGT TAT GGT GCG AGT AG	54°C
Lus10016121.g	GTG ACG TGG CCA AAG ATA A	CTC CCA TAG AGT AGC CAT ACA	54°C
Lus10039210.g	CTG TGT GCA AGT CGT GTA A	GGA AGG CTC ATC ATC AGT AAG	54°C
Lus10039511.g	GT GTC CCA CGG AAA TAT G	A CAC TTT CCA GGA AGC	54°C
Lus10041412.g	CAT GAA TCG TCC TGA TGT CC	CT TCC ACT CCC TCC TAA T	54°C

Three biological replicates (one replicate = six or seven pooled root samples) and three technical replicates for each biological replicate were used per treatment. The qRT-PCR reactions were performed on the CFX96 Real-Time System (BioRad). The CFX Manager Software (BioRad) program was used to calculate normalized expression values. To identify the significantly differentially expressed genes “R” (statistical computation R) packages were used: Univariate *post hoc* variance analysis (ANOVAs) was used to verify if there was significant difference between treatments; and Tukey’s (1949) honestly significant difference was subsequently used to identify which treatments presented significant differential gene expression when compared with the control (non-inoculated) (e.g. *F. oxysporum* f. sp. *lini*/control, *R. irregulare*/control, *F. oxysporum* f. sp. *lini-R. irregulare/*control) at the different time points.

## 3 Results

### 3.1 Phenotypic growth responses

#### 3.1.1 *Harvest 1* (9 dpi)

After nine dpi there were no differences among treatments in shoot length or total biomass (*n*=9-23, *Wilk’s λ*=0.87, *F*=1.80, *P*=0.13). Similarly, there were no differences detected at nine days among treatments in root parameters including root length, branching, or necrosis (*n*=3, *Wilk’s λ*=0.27, *F*=1.22, *P*=0.39).

#### 3.1.2 *Harvest 2* (14 dpi)

**Figure 1:**
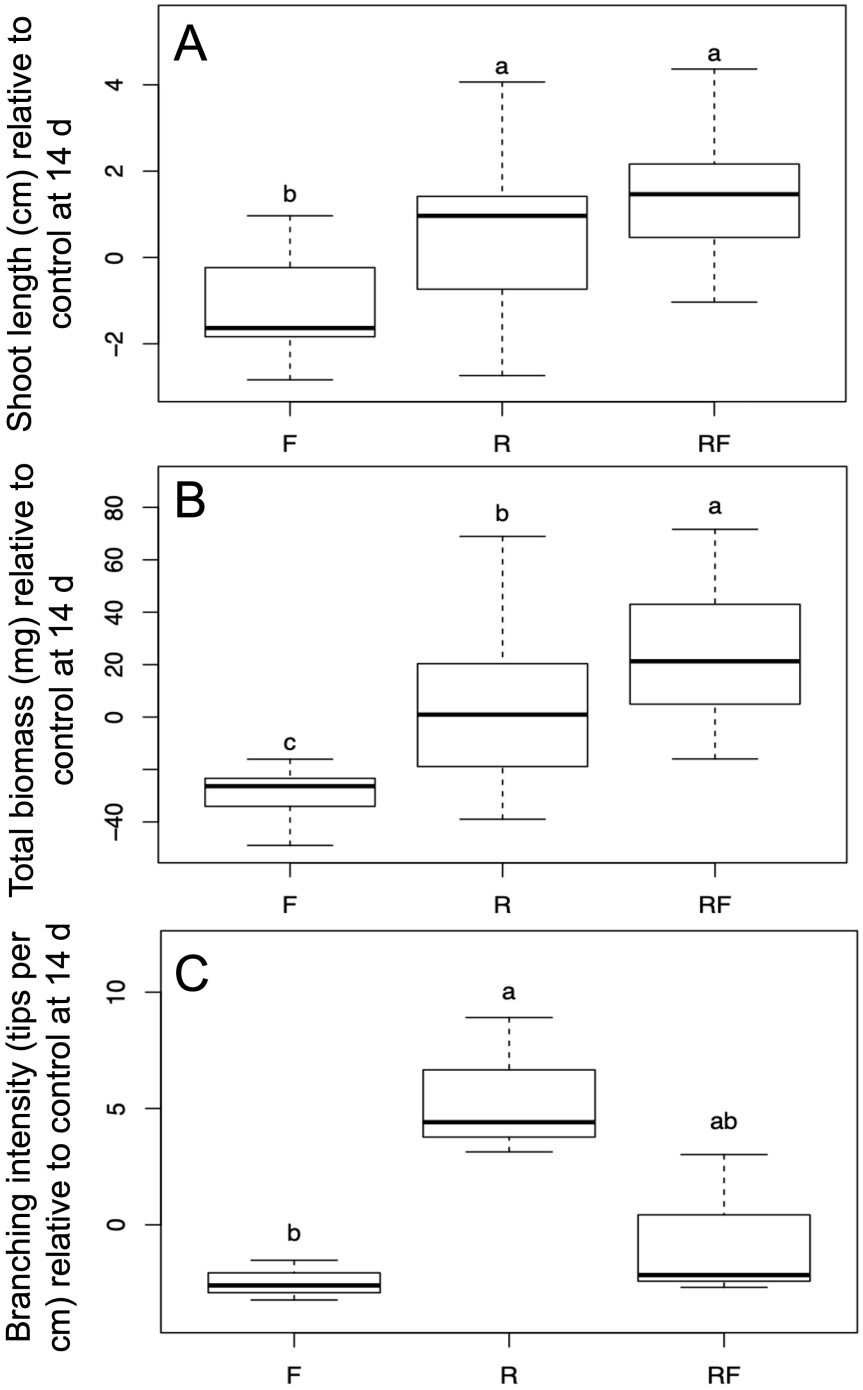
Effects of fungal treatments on flax seedling growth. **(A)** Shoot length relative to control after 14 days of growth (*n*=15-19). **(B)** Total biomass of flax plants relative to control after 14 days of growth (*n*=15-19). **(C)** Branching intensity in flax root systems relative to control Treatments (*n*=3). Treatments F, *Fusarium oxysporum (*f*)* sp. *lini*; R, *Rhizoglomus irregulare*; RF, *Rhizoglomus irregulare + Fusarium oxysporum (*f*)* sp. *lini.* Letters indicate significant differences according to Tukey’s honest significant difference (α=0.05).

After 14 dpi fungal treatment affected both shoot length and total biomass (*Wilk’s λ*=0.52, *F*=9.37, *P*<0.001). *Post-hoc* ANOVA and Tukey’s honest significant difference tests revealed that shoot length differed among treatments (*n*=15-19, *F*=12.24, *P*<0.001). Both the *R. irregulare* and *R. irregulare + F. oxysporum* f.sp*. lini* inoculated plants grew longer shoots relative to control than plants inoculated with *F. oxysporum* f.sp. *lini* alone (**Figure 1A**). Total biomass also differed among treatments (*n*=15-19, *F*=22.6, *P*<0.001). *R. irregulare* + *F. oxysporum* f.sp. *lini* inoculated plants not only produced more biomass than *F. oxysporum* f. sp. *lini* inoculated plants alone, they also produced more relative biomass than plants inoculated with *R. irregulare* alone (**Figure 1B**). Fungal inocula affected root parameters relative to controls as measured using WinRhizo (*n*=3, *Wilk’s λ*=0.10, *F*=2.88, *P*=0.08). Root length did not differ among treatments (*F=*1.78, *P=*0.24), nor did root necrotic surface area (*F=*3.05, *P=*0.12). However, relative branching intensity was affected by fungal treatment (*F=*7.81, *P=*0.02). Plants inoculated with *R. irregulare* produced root systems that were more branched than those inoculated with *F. oxysporum* f. sp. *lini*, while plants inoculated with both fungi showed similar branching intensity response to each fungus alone (**Figure 1C**). Other than the differences on growth responses between treatments, the plants did not show disease symptoms after up to 14 days of inoculation with the fungi (**Supplementary Figure 1**).

### 3.2 Root colonization assessment

*F. oxysporum* f. sp. *lini* was re-isolated from surface-sterilized roots of previously inoculated plants from the single and combined treatments at 9 and 14 dpi. Consistency in the morphology of colonies and spores when compared to the original inoculum, confirmed that the observed symptoms resulted from a fungal infection. Although the AMF fungus seemed to influence flax growth, especially in the *R. irregulare+F. oxysporum* f. sp. *lini* combination, we did not detect AMF growth inside the roots at either 9 or 14 dpi, despite extensive microscopic analysis.

### 3.3 RNA-seq

Following our observation that *F. oxysporum* f. sp. *lini* negatively affected flax growth parameters and that this negative effect was reversed when *R. irregulare* was co-inoculated with *F. oxysporum* f. sp. *lini* (**Figures 1A–C**), we used RNA-Seq to investigate the molecular responses to these two fungi individually and in combination. We compared the transcriptomes of four different treatments: non-inoculated control; *R. irregulare*; *F. oxysporum* f. sp. *lini*; and combined (co-inoculated with both fungi) (**Table 2**). Each treatment was sampled at two time points: 9 and 14 days post inoculation (dpi). Three independent pooled biological replicates were sequenced at each timepoint for each treatment. An average of 23.6 million reads was generated for each sample. An average of 92.8% of reads was mapped to the flax genome, indicating that the quality of the sequencing was sufficient for this analysis.

**Table 2:** RNA-Seq statistics.

Treatment	Days post-inoculation	Replicate	Total number of reads	Number of mapped reads	Mapped reads
*F. oxysporum* f. sp. *lini*	9	1	26,295,578	23,846,184	90.70%
	9	2	21,207,507	20,136,471	94.90%
	9	3	25,099,908	23,022,983	91.70%
	14	1	28,302,664	21,718,320	76.70%
	14	2	26,356,308	23,049,986	87.50%
	14	3	24,013,821	20,506,614	85.40%
water (non-inoculated)	9	1	26,296,784	25,614,703	97.40%
	9	2	28,154,058	27,107,516	96.30%
	9	3	23,256,284	21,423,289	92.10%
	14	1	26,290,590	25,555,104	97.20%
	14	2	27,218,277	25,835,470	94.90%
	14	3	23,948,405	22,394,920	93.5%
*R. irregulare*	9	1	26,297,248	25,672,917	97.60%
	9	2	27,085,471	25,915,966	95.70%
	9	3	47,990,672	43,327,088	90.30%
	14	1	26,296,505	25,548,569	97.20%
	14	2	27,960,824	26,790,071	95.80%
	14	3	25,439,847	24,738,422	97.20%
combined inoculum	9	1	26,840,322	25,709,225	95.80%
	9	2	25,444,731	24,919,946	97.9%
	9	3	25,459,037	24,889,473	97.8%
	14	1	24,728,927	23,493,166	95.00%
	14	2	28,124,855	26,849,645	95.50%
	14	3	25,447,103	24,826,809	97.60%
**Total**			566,160,856	525,222,951	N/A
**Average**			23,590,035	21,884,289	92.8%

### 3.4 Definition of differentially expressed genes

We calculated the normalized, relative transcript abundance (RNA-Seq fragments per thousand bases mapped, FPKM), averaged over all replicates for each treatment and time point. Transcript abundance was expressed as a log_2_ ratio of the treated sample relative to the non-inoculated control at each respective time point. Statistical significance was inferred as a false discovery rate (FDR, or q-value). In total, transcript abundance for 2,348 flax genes was significantly different (q < 0.05) from the non-inoculated control in one or more of the six treatments. In nearly all cases (94%; 2,215/2,348) in which a gene was defined as significantly different (q < 0.05), the absolute value of the log_2_ gene expression ratio was greater than 1, meaning that (in a linear scale), the gene had increased or decreased in abundance two or more fold. Therefore, to simplify further comparisons between treatments, and to avoid selecting an arbitrary expression ratio threshold, we decided to define all significantly different (q < 0.05) genes as differentially expressed genes (DEGs). Furthermore, any gene with a positive log_2_ gene expression ratio will be defined as “up-regulated”, and any gene with a negative log_2_ gene expression ratio will be defined as “down-regulated”, while acknowledging that we have here only measured transcript abundance, and not gene regulation *per se*. Tables of DEGs for each of the treatments are contained in the **Supplementary Data.**

### 3.5 Validation of RNA-seq data by qRT-PCR

We performed quantitative real-time PCR (qRT-PCR) reactions for 13 genes in all four treatments to assess the reproducibility of DEG measurements across platforms (**Tables 3**, **4**). The genes were selected to represent a variety of expression patterns, although the majority (9/13) were differentially expressed in the *F. oxysporum* f. sp. *lini* inoculated samples. The correlation between RNA-Seq and qRT-PCR results ranged from R= 0.8 at 9 dpi (**Table 3**) and R= 0.9 at 14 dpi (**Table 4**) for *F. oxysporum* f. sp. *lini* treatment, to R= 0.32 and R =0.18 for *R. irregulare* treatments. In general, genes that were defined as DEGs in each treatment had similar expression ratios in both RNA-Seq and qRT-PCR. The lower correlation among the *R. irregulare* treatments reflects the fact that fewer genes in these samples were defined as significantly different (q < 0.05) in the RNA-Seq experiment (see below). Overall, these observations provide evidence the RNA-Seq results we present here are generally valid.

**Table 3:** Comparison of RNA-Seq and qRT-PCR log2 expression ratios (treatment/control) for selected genes at 9 dpi.

Lus id	annotation	9 dp						
		*F. oxysporum* f. sp. *lini*		*R. irregulare*		combined		
		RNA- Seq	qRT- PCR	RNA-Seq	qRT- PCR	RNA-Seq	qRT- PCR	
10002741	LTP/seed storage 2S albumin	3.0*	4.4*	-2.2*	-1.7	-0.4	-1.6
10004410	PR-related thaumatin superfamily	9.2*	5.1*	-2.6	-0.3	4.9*	-0.2
10005358	spermidine hydroxycinnamoyl trfase.	7.1*	6.0*	-1.7	-0.6	2.2*	-0.5
10005858	alpha/beta-Hydrolases superfamily	3.7*	2.6	-1.0	-0.4	0.4	-0.4
10006691	O-methyltransferase family	5.5*	2.8	-3.3*	-0.5	1.0	-0.1
10009254	germin-like protein 10	-11.6	-1.7	1.5	-0.2	-0.1	0.2
10010696	nodulin MtN21 transporter family	0.1	-1.3	1.5*	2.4	0.1	-0.3
10012880	terpenoid cyclase superfamily	4.4*	4.7*	-0.9	-1.3	0.4	-1.2
10014241	MLP-like protein 423	-2.2*	-6.5*	-3.0*	-7.1*	-3.6*	-7.4*
10016121	nitrate transporter 2:1	0.1	-1.5	1.9*	2.1	1.6*	0.8
10039210	Kunitz family trypsin/protease inhibitor	10.5	8.6*	1.8	-0.4	5.3	-1.0
10039511	LTP/seed storage 2S albumin	6.3*	7.3*	-0.2	-1.0	0.8	0.7
10041412	serine carboxypeptidase-like 28	-6.2	-2.1	7.4	-1.5	5.4	-1.0
		*R*=0.8		*R*=0.32		*R*=0.51		
		*p* =0.001		*p*=0.29		*p* =0.078		

Values shown are log2 scale expression ratio (treatment/control). Lus id is the numeric portion of the flax gene identifier (e.g. Lus10002741) in Phytozome. The fungal species used for each inoculation are as named, except “combined” which is the simultaneous inoculation. Data shown are the average of three replicates in RNA-Seq and three replicates in qRT-PCR. The asterisk (*) and bold typeface denotes statistical significance (q <0.05, for RNA-Seq) and (p <0.05) for qRT-PCR.

**Table 4:** Comparison of RNA-Seq and qRT-PCR log2 expression ratios (treatment/control) for selected genes at 14 dpi.

Lus id	annotation	14dpi						
		*F. oxysporum* f. sp. *lini*		*R. irregulare*		combined		
		RNA-Seq	qRT-PCR	RNA-Seq	qRT-PCR	RNA-Seq	qRT-PCR	
10002741	LTP/seed storage 2S albumin	2.7*	3.0*	-0.6	-0.5	0.8*	0.9	
10004410	PR-related thaumatin superfamily	11.2*	8.4*	-0.8*	0.2	8.2*	4.0*	
10005358	spermidine hydroxycinnamoyl trfase.	**8.3**	**9.7**	0.6	0.6	**5.4**	**4.5**	
10005858	alpha/beta-Hydrolases superfamily	6.1*	7.8*	0.4	0.5	3.1*	1.2	
10006691	O-methyltransferase family	4.3*	4.2*	-3.7*	-0.3	2.4*	1.9	
10009254	germin-like protein 10	-3.8	-3.9	-2.3	0.5	-0.8	-0.3	
10010696	nodulin MtN21 transporter family	-1.9	-0.5	0.2	0.9	1.1*	0.8	
10012880	terpenoid cyclase superfamily	2.8	5.6*	0.1	-1.3	2.1	5.6*	
10014241	MLP-like protein 423	-1.9	-4.1*	-4.5*	-5.9*	-3.2	-5.8	
10016121	nitrate transporter 2:1	-2.8*	-0.1	0.8	0.1	1.5	0.2	
10039210	Kunitz family trypsin/protease inhibitor	8.0*	7.0*	-0.5	-0.8	3.8	3.7*	
10039511	LTP/seed storage 2S albumin	8.1*	13.7*	-0.5	-1.2	4.8	-1.7	
10041412	serine carboxypeptidase-like 28	0.1	-2.4	-9.2	0.8	-10.7	-0.7	
		*R*=0.9		*R*=0.18		*R*=0.54		
		*p* =2.4 x 10^-5^		*p*=0.57		*p* =0.054		

Values shown are log2 scale expression ratio (treatment/control). Lus id is the numeric portion of the flax gene identifier (e.g. Lus10002741) in Phytozome. The fungal species used for each inoculation are as named, except “combined” which is the simultaneous inoculation. Data shown are the average of three replicates in RNA-Seq and three replicates in qRT-PCR. The asterisk (*) and bold typeface denotes statistical significance (q <0.05, for RNA-Seq) and (p <0.05) for qRT-PCR.

### 3.6 Visualization and clustering of DEGs

**Figure 2:**
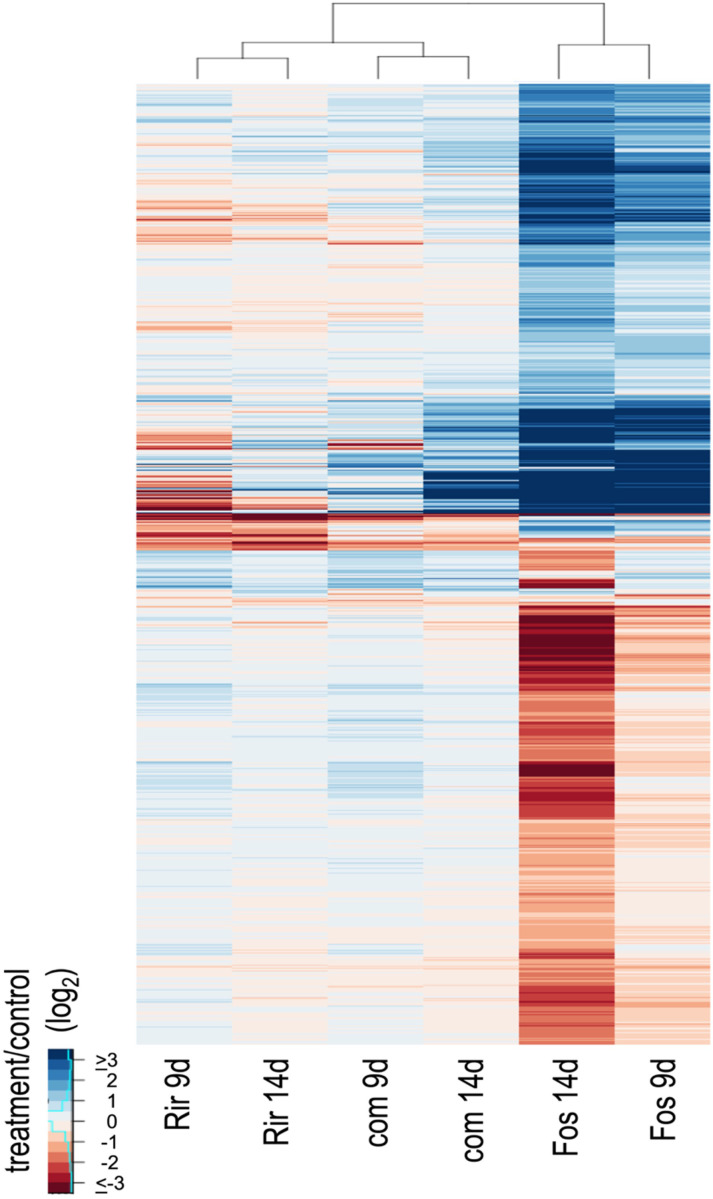
Hierarchical clustering and heatmap of expression ratios (log2, treatment/control) for 2,348 DEGs. Treatments are *R. irregulare* (Rir), *F. oxysporum* f. sp. *lini* (Fos) or the combined inoculum (com) after 9 or 14 dpi, compared to a non-inoculated control at the same time point. Hierarchical clustering was applied to both rows and columns of the heatmap.

The 2,348 DEGs were visualized using a heat map and hierarchical clustering (**Figure 2**). The heat map showed that the transcriptome response differed more between inoculum types than between the time points sampled. Furthermore, the responses to *R. irregulare* and the combined inoculum were more similar to each other than either was to *F. oxysporum* f. sp. *lini*. The heatmap also showed that the *F. oxysporum* f. sp. *lini* 14 dpi treatment was overwhelmingly the treatment that induced the highest number of responsive genes (2,117).

### 3.7 Principal component analysis of all treatments

**Figure 3:**
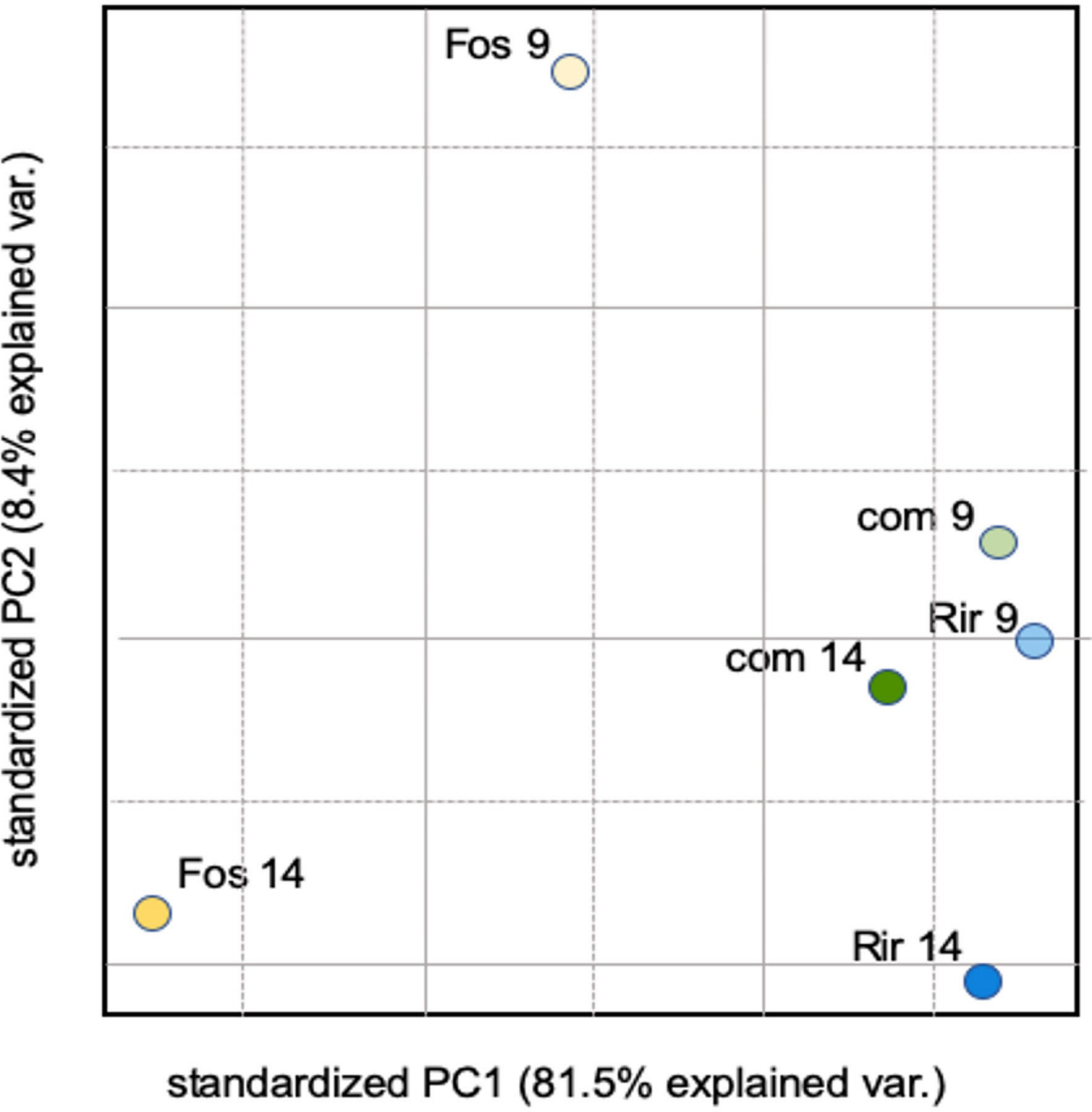
PCA of average log_2_ expression ratios (treatment/control) for 2,348 DEGs. Treatments are *R. irregulare* (Rir), and *F. oxysporum* f. sp. *lini* (Fos), or both fungi combined (com) at 9 or 14 dpi.

We conducted Principal Component Analysis (PCA) of the 2,348 DEGs expressed genes to facilitate the identification of patterns and relationships among the transcriptome responses to each treatment (**Figure 3**). The two major principal components together explained 89.9% of the total variance, and showed that the *R. irregulare* 9 dpi and the combined treatments were most similar, and these were much more similar to *R. irregulare* 14 dpi than to *F. oxysporum* f. sp. *lini* at either 9 dpi or 14 dpi. A similar result was obtained when the expression ratios of all genes (not just those with q < 0.05) were used to conduct PCA (data not shown).

### 3.8 Global patterns of gene expression of all treatments

**Figure 4:**
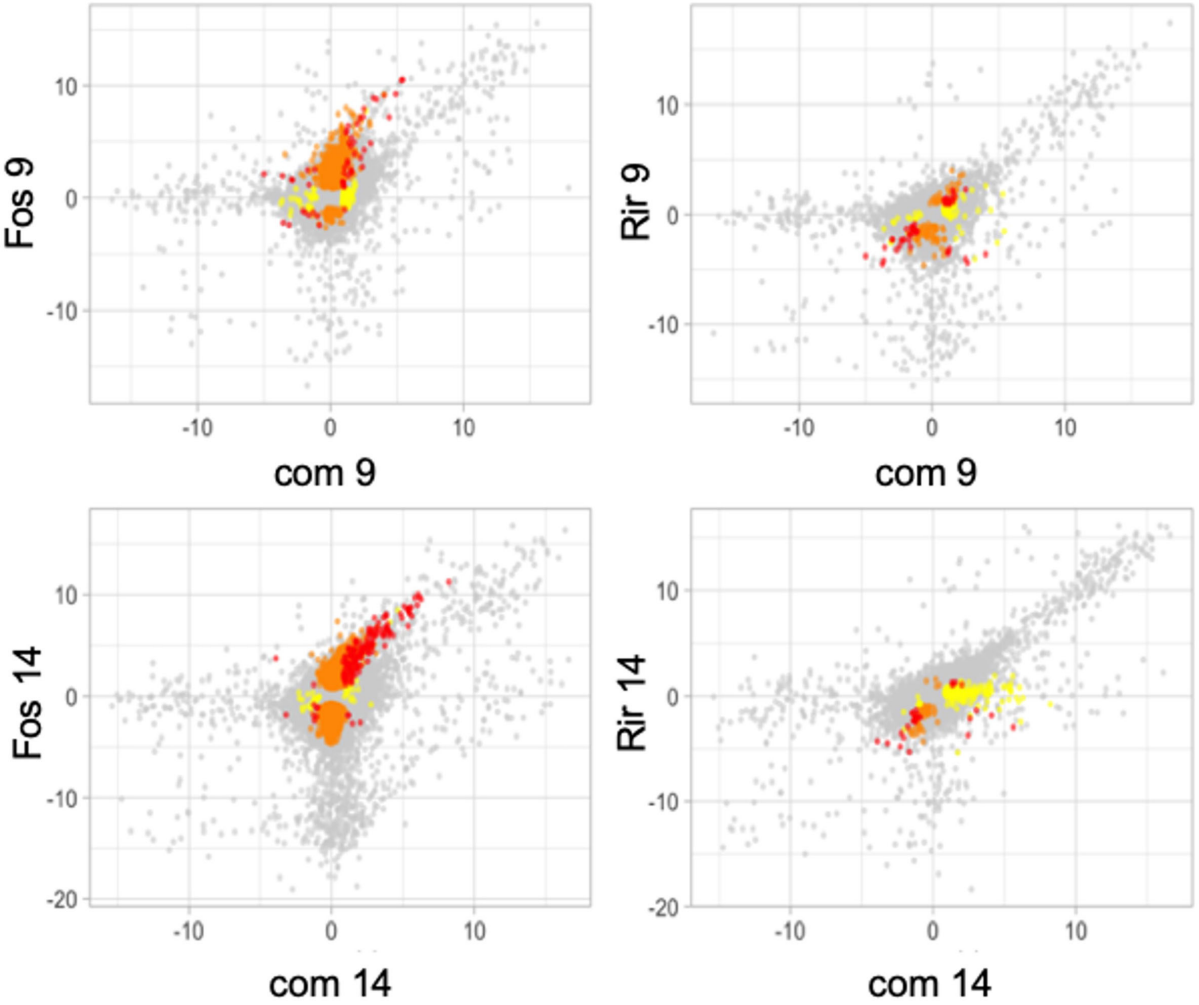
Scatter plots of average expression ratios (log2, treatment/control) for all 32,807 flax genes measured by RNA-Seq experiments. Genes that were significantly differentially expressed (q<0.05) compared to control in both of the treatments in the graph are red, genes that are significantly differentially expressed in only the treatment on the y-axis are orange, and genes significantly differentially expressed in only the treatment shown on the x-axis are yellow, and genes that were not significantly different from controls in either treatment are grey. Treatments represented are *R. irregulare* (Rir), and *F. oxysporum* f. sp. *lini* (Fos), or both fungi combined (com) after 9 or 14 days of treatment. Data are the average of three independent replicates.

The effect of inoculation with a single fungal species as compared to both fungal species simultaneously was shown when gene expression ratios were plotted for all 32,807 flax genes (**Figure 4**). These plots show that genes that have large, positive, expression ratios are more likely than genes with large, negative, expression ratios to have conserved expression patterns in the combined inoculum as compared to the individual inocula. This observation was particularly evident in the comparisons involving *F. oxysporum* f. sp. *lini* 14 at dpi.

### 3.9 Identification of DEGs responsive to inoculation by either *F. oxysporum* f. sp. *lini* or *R. irregulare*

**Figure 5:**
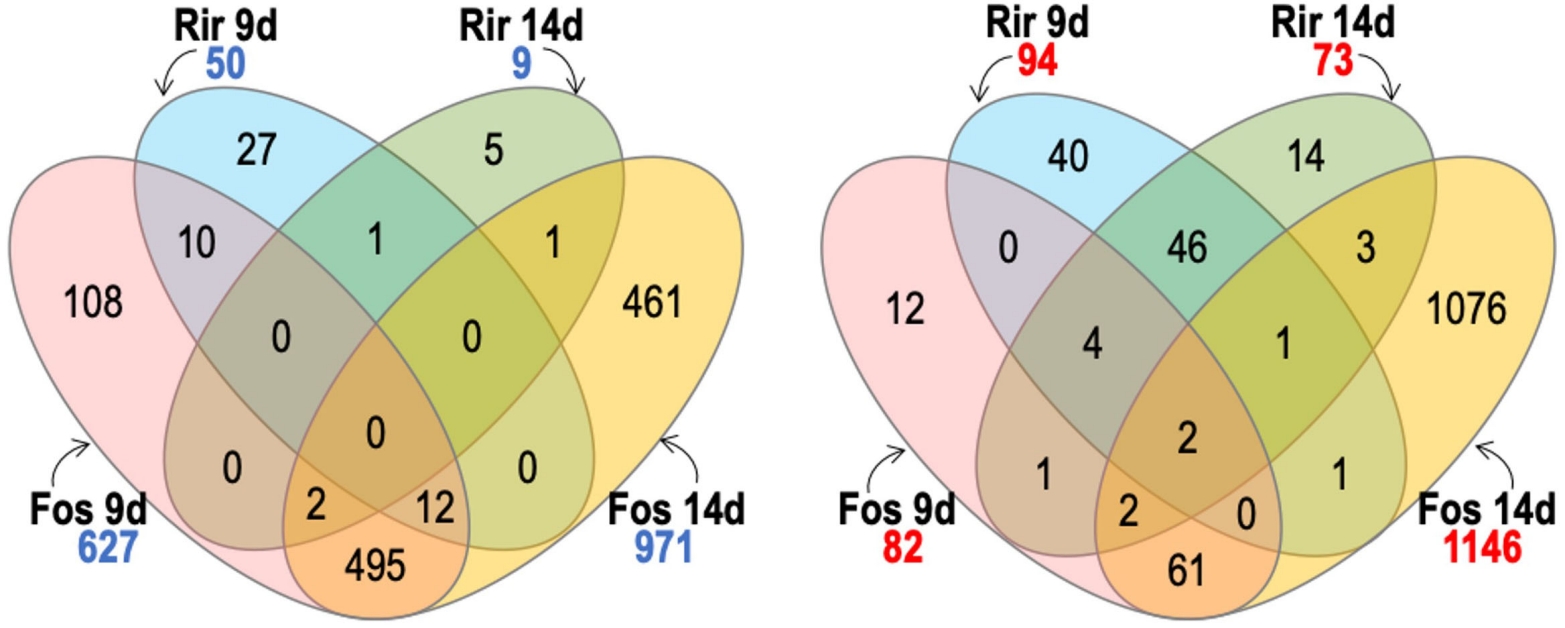
Venn diagram comparing the number of significant transcripts with significantly increased abundance (numbers in blue) and transcripts with significantly decreased abundance (numbers in red) of flax roots inoculated with *R. irre gulare* (Rir) or *F. oxysporum* f. sp. *lini* (Fos), at 9 and 14 days post infection. We considered significant differential expression between samples and non-inoculated control when FDR < 0.05, and fold change >0 (transcripts with increased abundance) or <0 (transcripts with decreased abundance).

We used Venn diagrams to compare and contrast groups of DEGs that were responsive to our various treatments. As shown in **Figure 5**, *F. oxysporum* f. sp. *lini* 14 dpi was the treatment with the highest number of up-regulated (971) or down-regulated (1146) genes, as was also evident from the heatmap (**Figure 2**). *F. oxysporum* f. sp. *lini* also resulted in a large number (627) of up-regulated genes at 9 dpi, and most of these (81%; 509/627) were also up-regulated at 14 dpi. However, a relatively smaller number of genes (82) were down-regulated by *F. oxysporum* f. sp. *lini* at 9 dpi.

The *R. irregulare* treatment produced 50 up-regulated genes at 9 dpi, and only 9 up-regulated genes 14 dpi. This was far fewer than the number of genes up-regulated by *F. oxysporum* f. sp. *lini* at either time point. The number of genes down-regulated (73) by *R. irregulare* 14 dpi was also comparatively low, but the number of genes down-regulated (94) by *R. irregulare* at 9 dpi was higher than in *F. oxysporum* f. sp. *lini* at 9 dpi. The most overlap between different treatments was found among the genes up-regulated at 9 dpi, where we observed that 44% (22/50) of the genes that were up-regulated in 9 dpi *R. irregulare* treated plants were also up-regulated in 9 dpi *F. oxysporum* f. sp. *lini* treated plants. There was otherwise very little overlap between the genes that responded to *F. oxysporum* f. sp. *lini* and the genes that responded to *R. irregulare.*

### 3.10 GO Functional Enrichment – Functions of *F. oxysporum* f. sp. *lini* DEGs

We used Gene Ontology (GO) enrichment analysis to characterize the functions of DEGs responsive to treatments (**Table 5**; **Supplementary Table S1**). This analysis identified over 246 significantly enriched GO terms, highlighting key molecular functions, biological processes and cellular components affected by experimental conditions (**Table 5**).

**Table 5:** Gene Ontology (GO term) enrichment analysis for DEGs.

GO Term		description	*F. oxysporum* f. sp. *lini*				*R, irregulare*				combination			
			9 dpi		14 dpi		9 dpi		14 dpi		9 dpi		14dpi	
			dn	up	dn	up	dn	up	dn	up	dn	up	dn	up
0009694	P	jasmonic acid metabolic proc		6										
0009627	P	systemic acquired resistance		5										
0009620	P	response to fungus		14		13								
0030246	F	carbohydrate binding		8		10								
0016614	F	oxidoreductase, acting on CH-OH		8		14								
0051119	F	sugar transmembrane transporter		9		9								
0048046	C	apoplast			14									
0005975	P	carbohydrate met proc		21	37									
0034641	P	cellular N compound met proc		14	22									
0009755	P	hormone-mediated sig path		11	18									
0008610	P	lipid biosynthetic process		14	24									
0004601	F	peroxidase activity		7	16	11								
0008194	F	UDP-glycosyltransferase activity		9	16	14								
0009753	P	response to JA stimulus		15	13	15								
0006629	P	lipid met proc		22	41							7		
0032787	P	monocarboxylic acid met proc		17	25	18						5		
0009699	P	phenylpropanoid biosyn proc		10	20	11						6		
0019438	P	aromatic compound biosyn proc		12	24	13						7		
0006575	P	cellular aa derivative met proc		14	26	15						8		
0009698	P	phenylpropanoid met proc		10	21	13						6		
0022857	F	transmembr transporter activity		28	43	37						11		10
0006955	P	immune response		18		22	10		6					8
0006952	P	defense response		40		46	10		6			6		12
0005618	C	cell wall	6	18	45	24						5		8

GO term enrichment among DEGs, calculated GSEA analysis in AgriGO. The GO terms shown are from each of three GO domains: C (cellular component), F (molecular function), or P (biological process). Up-regulated DEGs (up) are shown in blue; and Down-regulated DEGs (dn) are shown in red. Values in colored boxes show the number of genes assigned to each GO term. Values are shown only for GO terms for which enrichment was statistically significant (FDR < 0.05). process (proc), metabolism (met), signaling (sig), pathway (path). This table shows only selected GO terms; the full table can be found in the **Supplementary Table S1.**

The majority of the enriched categories were identified as a result of *F. oxysporum* f. sp. *lini* inoculation, as it had the largest number of DEGs (**Table 5**). Inoculation with the pathogenic fungus was associated with categories related to defense (e.g. jasmonic acid (JA) metabolism, systemic acquired resistance, and response to fungus) at 9 dpi, and metabolism and signaling (e.g. carbohydrate binding, oxidoreductase, and sugar transmembrane transporter) at both time points. These categories were not enriched by other treatments. Only the category apoplast was uniquely enriched among *F. oxysporum* f. sp. *lini* down-regulated DEGs at 14 dpi. These apoplast-category genes are likely involved in cell wall modifications. Common enrichments among up and down-regulated genes in response to only *F. oxysporum* f. sp. *lini* alone include various metabolic processes (e.g. carbohydrate, and lipid), hormone-mediated signaling (e.g. jasmonic acid), enzymatic activities (peroxidase and UDP-glycosyltransferase), among others (**Table 5**). Common enrichments among *F. oxysporum* f. sp. *lini* DEGs in the alone and combined treatment consisted of categories of genes related to metabolism and secondary metabolites (**Table 5**). Finally, we observed an interesting contrast between response to inoculation with *F. oxysporum* f. sp. *lini* (alone and combined inocula) and inoculation with *R. irregulare* alone, as the categories immune and defense response were enriched only following inoculations involving the pathogenic fungus.

### 3.11 GO Functions of *R. irregulare* responsive DEGs

Although no GO terms were uniquely enriched in DEGs in response to *R. irregulare* inoculation, functional groups were evident in DEGs at both time points (**Tables 6**, **7**; **Supplementary Tables S2**, **S3**). Down-regulated DEGs included heavy metal transporters, transcription factors, major-latex proteins (MLPs); various pathogenesis related (PR) proteins, and multiple chitinases and beta-1,3-glucanases (**Table 7**).

**Table 6:** Comparison of genes up-regulated by *R. irregulare* at any time point that were oppositely down-regulated by *F. oxysporum* f. sp. *Lini*.

Lus id	annotation	F. oxysporum f. sp. lini		R. irregulare		combined	
		9 d	14 d	9 d	14 d	9 d	14 d
10039259	Ca-dependent lipid-binding family	0.13	**-1.50**	**1.30**	0.14	**0.88**	0.29
10031145	cytochrome P450 79B2	0.62	**-4.46**	**1.41**	0.59	**1.39**	0.01
10009152	Gln-amidotransferase-like superfamily	-0.20	**-1.70**	**1.06**	-0.07	0.39	-1.35
10033360	hydroxyproline-rich glycoprotein	0.60	**-1.59**	**1.39**	0.25	**1.10**	0.79
10039881	hydroxyproline-rich glycoprotein	-0.63	**-4.48**	**1.29**	-0.02	1.03	0.23
10030872	K^+^ transporter, high affinity	0.08	**-1.94**	**1.30**	-0.03	0.89	0.27
10022066	LTP/seed storage 2S albumin superfamily	0.43	**-1.94**	**1.15**	0.40	**1.28**	0.68
10016121	NO_3_^-^ transporter	0.12	**-2.76**	**1.89**	0.75	**1.61**	**1.46**
10010696	nodulin MtN21 transporter family	0.10	**-1.91**	**1.47**	0.19	0.13	**1.11**
10018628	O-methyltransferase family	-0.29	**-1.86**	**1.30**	0.17	0.44	0.69
10025256	peroxidase superfamily	0.16	**-2.71**	**1.40**	0.54	**1.57**	0.58
10006534	peroxidase superfamily	**-2.21**	**-2.65**	-0.20	**1.07**	0.92	**2.01**
10001228	peroxidase superfamily	0.66	**-2.46**	**1.14**	0.28	**1.35**	-0.07
10008213	pollen Ole e1 allergen, extensin family	0.43	**-2.83**	**1.05**	**0.92**	**1.22**	0.24
10024122	polyphenol oxidase	0.59	**-1.63**	**1.05**	0.59	**1.09**	0.29
10011335	senescence-associated gene 29	0.34	**-2.44**	**1.18**	-0.14	0.37	-0.55
10038848	unknown function	0.22	**-2.35**	**1.06**	0.64	**1.07**	0.69
10003550	unknown function	0.58	**-1.68**	**1.20**	0.19	0.74	0.58
10010793	unknown function (DUF607)	-0.72	**-1.37**	**1.14**	0.32	0.23	0.16
10018645	unknown function (PELPK like)	0.24	**-2.47**	**1.16**	0.10	0.98	0.50

The asterisk (*) and bold typeface denotes statistical significance (q <0.05, for RNA-Seq) and (p <0.05) for qRT-PCR.

**Table 7:** Comparison of genes down-regulated by *R. irregulare* at any time point that were oppositely up-regulated by *F. oxysporum* f. sp. *lini*.

Lus id	annotation	*F. oxysporum* f. sp. *lini*		*R. irregulare*		combined	
		9 d	14 d	9 d	14 d	9 d	14 d
10022191	2-oxoglutarate (2OG), Fe(II)-dependent oxygenase	**4.48**	**6.61**	**-2.08**	0.52	0.07	**3.62**
10004364	alpha/beta-hydrolases superfamily	0.27	**1.22**	**-1.97**	**-1.83**	-0.95	-0.36
10038524	alpha/beta-hydrolases superfamily	**1.57**	**3.65**	**-1.43**	-0.30	0.85	0.08
10019801	beta-1,3-glucanase 1	7.77	**7.85**	**-4.39**	-2.99	**2.66**	**5.62**
10018696	C2H2 and C2HC zinc fingers superfamily	0.91	**1.45**	**-1.47**	-0.86	-0.09	-0.35
10020686	Calmodulin-binding protein	-0.24	**1.12**	**-1.80**	**-2.13**	-0.30	**-1.25**
10003231	chitinase, homolog of carrot EP3-3	**6.42**	**7.25**	**-3.15**	**-1.86**	**1.31**	**4.10**
10010866	chitinase, homolog of carrot EP3-3	**3.49**	**5.23**	**-1.88**	0.42	-0.48	**2.41**
10024366	chitinase, homolog of carrot EP3-3	**5.95**	**6.48**	**-3.12**	-1.38	0.73	**3.02**
10028275	chitinase, homolog of carrot EP3-3	**1.74**	**2.43**	**-1.10**	**-1.07**	0.08	0.03
10016464	copper transporter 1	0.91	**0.92**	-0.84	**-1.13**	-0.47	-0.37
10014284	cytochrome BC1 synthesis	**1.08**	**2.25**	-1.11	**-1.42**	0.33	0.13
10030189	cytochrome P450 71B10	**2.36**	**2.88**	**-1.27**	0.39	-0.79	0.88
10013150	cytochrome P450 82C	**1.34**	**1.28**	**-0.84**	-0.80	-0.29	0.06
10038200	cytochrome P450 82C4	**3.21**	**5.92**	**-1.69**	1.23	0.12	**3.99**
10011658	cytochrome P450 82C4	**2.53**	**4.52**	**-1.71**	0.67	**-1.19**	**1.54**
10025901	cytochrome P450 82C4	**3.20**	**4.74**	**-1.78**	0.63	-0.39	**2.19**
10025902	cytochrome P450 82C4	**5.30**	**7.67**	**-2.23**	0.88	0.92	**4.93**
10010742	extensin-like	**2.14**	**3.70**	**-3.78**	**-4.30**	**-5.01**	**-3.91**
10030362	glutathione S-transferase TAU 24	0.31	**2.55**	**-1.72**	0.49	-0.87	**1.41**
10033420	integrase-type DNA-binding superfamily	-0.81	**1.67**	**-4.61**	**-1.72**	-0.63	-0.39
10002741	LTP/seed storage 2S albumin superfamily	**3.04**	**2.74**	**-2.17**	-0.62	-0.36	0.83
10016323	LTP/seed storage 2S albumin superfamily	**2.24**	**2.24**	**-1.45**	-0.67	-0.40	0.54
10022642	LYS/HIS transporter 7	**1.63**	**2.31**	**-1.42**	-0.60	-0.74	**1.32**
10032976	malectin/receptor-like protein kinase	**1.32**	**1.45**	**-1.16**	-0.27	-0.26	-0.01
10005605	matrixin family	**3.46**	**3.18**	**-3.78**	-0.85	0.17	**0.98**
10011820	myb domain protein 15	**1.11**	**2.07**	**-1.07**	**-1.19**	-0.06	-0.44
10024795	N-terminal nucleophile aminohydrolase	2.73	**4.58**	**-1.17**	0.46	0.20	**1.18**
10038332	NAC domain containing protein 42	**2.78**	**4.27**	**-2.28**	1.24	-1.14	**2.07**
10029369	NAD(P)-binding Rossmann-fold superfamily	**3.85**	**5.70**	**-2.19**	0.50	0.20	**2.52**
10020783	NDR1/HIN1-like 25	0.83	**1.87**	**-1.38**	-0.17	0.35	0.15
10041460	NIM1-interacting 2	**2.50**	**2.21**	**-2.39**	**-2.52**	-1.74	0.31
10006691	O-methyltransferase family protein	**5.47**	**4.32**	**-3.35**	**-3.73**	1.03	**2.44**
10040215	ortholog of sugar beet HS1 PRO-1 2	0.66	**1.31**	-1.05	**-0.95**	-0.06	-0.31
10006302	osmotin 34 PR5	**5.80**	**4.94**	**-3.43**	**-2.17**	1.17	**2.59**
10017170	osmotin 34 PR5	**5.34**	**5.27**	**-3.75**	-0.68	0.86	**2.63**
10035775	P-loop containing NTP hydrolases superfamily	**1.22**	**1.30**	**-2.10**	**-1.55**	-1.08	-0.21
10032178	pathogenesis-related Bet v 1	**5.99**	**8.69**	**-3.55**	0.42	**1.21**	**5.45**
10015339	pathogenesis-related Bet v 1	4.46	**5.87**	**-2.27**	0.56	0.25	**3.03**
10020493	pathogenesis-related PR1	9.17	**8.97**	**-3.61**	-0.51	**4.03**	**6.01**
10012479	pathogenesis-related PR1	**7.93**	**9.54**	**-4.04**	-0.17	**2.51**	**6.29**
10012684	peroxidase superfamily	**3.32**	**3.88**	**-2.60**	-0.25	0.39	**1.81**
10000327	polynucl. transferase, RNAse H-like super fam.	0.08	**1.57**	**-2.46**	**-1.67**	0.42	-0.86
10035078	polynucl. transferase, RNAse H-like super fam.	-0.22	**1.12**	**-2.19**	**-1.58**	0.44	-0.86
10003116	RmlC-like cupins superfamily	**1.02**	**1.72**	**-1.01**	0.84	-0.63	-0.62
10032848	salt tolerance zinc finger	-0.27	**1.47**	**-2.53**	**-1.39**	0.03	-0.72
10038985	sigma factor binding protein 1	**1.16**	0.87	**-2.99**	**-2.63**	**-1.62**	-1.03
10027279	sigma factor binding protein 1	**1.73**	0.74	**-2.37**	**-2.73**	-1.21	-1.00
10008742	UDP-glycosyltransferase 74 F1	1.17	**3.26**	**-2.00**	0.91	-0.37	0.48
10034389	unknown function	**2.52**	**3.13**	**-1.68**	-1.17	-0.89	0.14
10032881	unknown function (DUF 1645)	0.48	**1.43**	**-1.07**	-0.67	0.17	-0.39
10037249	unknown function (DUF 4228)	-0.10	**1.25**	**-1.13**	-0.25	0.17	0.24
10024074	WRKY DNA-binding protein 40	**1.17**	**2.08**	**-1.19**	**-1.54**	0.78	-0.56
10034244	WRKY DNA-binding protein 70	**1.72**	**2.05**	**-1.75**	**-1.75**	-0.49	0.07
10012870	WRKY DNA-binding protein 70	**0.93**	**1.24**	**-1.14**	-1.61	-0.54	-0.37

Table shows expression ratio (log2) for treatment:control. Genes shown have FDR < 0.05 in at least one of the treatments.The asterisk (*) and bold typeface denotes statistical significance (q <0.05, for RNA-Seq) and (p <0.05) for qRT-PCR.

Fewer up-regulated genes included transporters like nodulin MtN21 high-affinity K+ transporter, nitrate transporter and phosphate transporter 1;3, alongside cell wall and polysaccharide-modifying proteins (**Supplementary Table 2**).

### 3.12 DEGs with opposite responses to *F. oxysporum* f. sp. *lini* and *R. irregulare*

**Figure 6:**
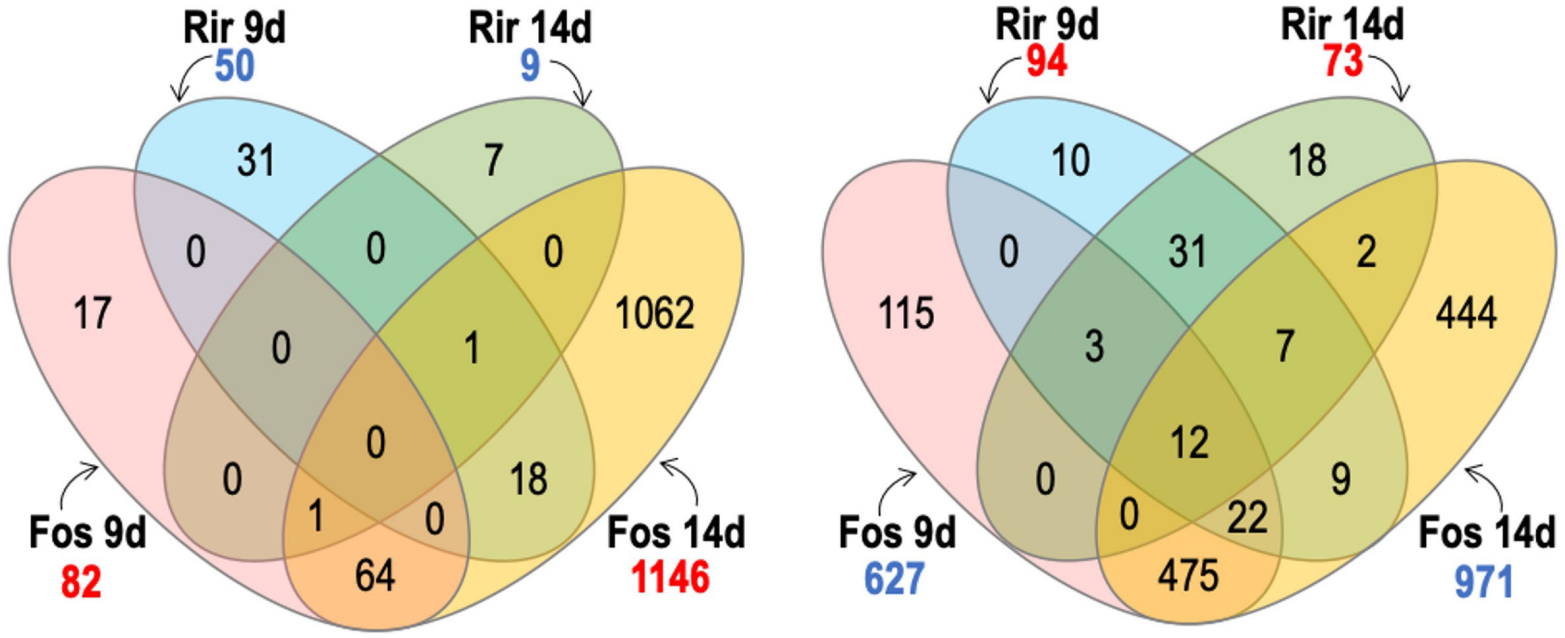
Venn diagram shows contrasts between genes with significant transcripts with significantly increased abundance (numbers in blue) and transcripts with significantly decreased abundance (numbers in red) of flax roots inoculated with *R. irregulare* (Rir) or *F. oxysporum* f. sp. *lini* (Fos).We considered significant differential expression between samples and non-inoculated control when FDR < 0.05, and fold change >0 (transcripts with increased abundance) or <0 (transcripts with decreased abundance).

Because we hypothesized that some genes of interest might respond differently to a pathogen (*F. oxysporum*) than a mutualist (*R. irregulare*), we used Venn diagrams to represent sets of genes that were up-regulated by one fungus, and down-regulated by the other (**Figure 6**). From the few genes up-regulated by *R. irregulare* (59 in total), 20 were oppositely down-regulated by *F. oxysporum* f. sp. *lini* (**Table 6**). These include nutrient transporters, cell wall remodeling and peroxidases and might be critical for mutualism. A slightly higher number of genes were down-regulated by *R. irregulare* with opposite regulation by *F. oxysporum* f. sp. *lini* (**Table 7**). These included many defense genes, including transcription factors, chitinases, pathogenesis-related proteins (PR), and others. This result suggests that the suppression of defense genes might be required for mutualism.

### 3.13 Simultaneous inoculation with a combination of *F. oxysporum* f. sp. *lini* and *R. irregulare*

**Figure 7:**
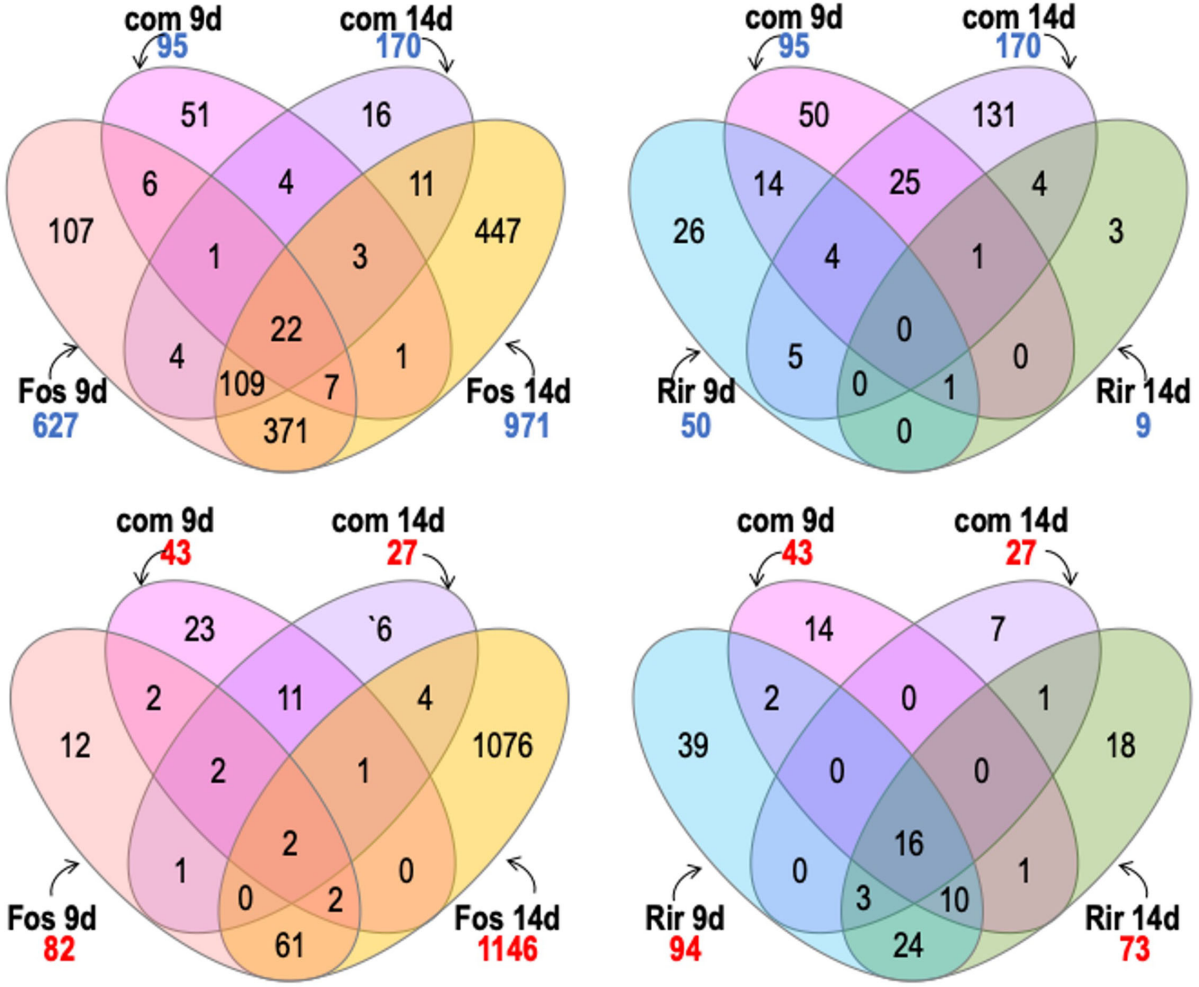
Venn diagram shows comparisons between genes with significant transcripts with increased abundance (numbers in blue) and transcripts with significant decreased abundance (numbers in red) of flax roots inoculated with the combined inoculum (*R. irregulare* and F*. oxysporum* f. sp. *lini*) compared to inoculation with *R. irregulare* (Rir) or *F. oxysporum* f. sp. *lini* (Fos) alone. We considered significant differential expression between samples and non-inoculated control when FDR < 0.05, and fold change >0 (transcripts with increased abundance) or <0 (transcripts with decreased abundance.

Treatments with the combined inoculum (*R. irregulare* and *F. oxysporum* f. sp. *lini)* showed 95 genes increased in transcript abundance at 9 dpi, and 170 genes increased in transcript abundance at 14 dpi (**Figure 7**). At either time point, a much smaller number of genes increased in response to the combined inoculum compared to the 627 or 971 genes that increased at 9 and 14 dpi respectively, in response to *F. oxysporum* f. sp. *lini* alone. The response to the combined inoculum was therefore not simply the sum of responses to the individual inocula. However, most of the 170 genes that did increase following the combined 14 dpi treatment were also induced by *F. oxysporum* f. sp. *lini* alone at either 9 dpi (136/170) or 14 dpi (145/170). Thus, most of the transcripts induced in the combined inoculum at 14 dpi were also induced by *F. oxysporum* f. sp. *lini* alone (at both 9 dpi and 14 dpi), although these common transcripts are only a small fraction (145/971) of the total transcriptomic responses to *F. oxysporum* f. sp. *lini* alone at 14 dpi. In contrast, when the genes commonly increased in *R. irregulare* and the combined inoculum at 9 dpi are compared, only 20% (19/95; 9 dpi) or 5% (9/170; 14 dpi) were commonly induced by both types of treatment.

Many of the DEGs with the highest expression (log_2_ fold change > 4) in the combined treatment at 14 dpi were also strongly expressed in the *F. oxysporum* f. sp. *lini* alone treatment, including: two PR1 genes (Lus10004410 and Lus10012479) and one homolog of carrot EP3-3 chitinase (Lus10003231).

The presence of *R. irregulare* in the combined inoculum resulted in the suppression (or non-activation) of some genes that were up-regulated by exposure to *F. oxysporum* f. sp. *lini* alone. The most prominent of these were: Kunitz family trypsin and protease inhibitor protein (Lus10007888, Lus10007889, Lus10022302, Lus10026357, Lus10039209, and Lus10039210), and plant invertase/pectin methylesterase inhibitor superfamily Lus10038917, Lus10027202 and Lus10031133) (**Supplementary Tables S6**, **S7**).

Other genes were completely repressed by *R. irregulare* in the combined treatment in comparison with the *F. oxysporum* f. sp. *lini* treatment alone, including: transcription factors (Lus10024074, Lus10034244 and Lus10012870); other signaling proteins such as NDR1/HIN1-like 25 (Lus10020783) and MAP kinases (Lus10025986, Lus10040127, Lus10040128, and Lus10001081); calmodulin-binding protein (Lus10020686, Lus10008742), genes related to JA pathway (Lus10027648 and Lus10039911) (**Supplementary Tables S6**, **S7**).

Decreases in transcript abundance were observed for 43 genes at 9 dpi, and only 27 genes at 14 dpi following the combined inoculum treatment. Of these, 19% (8/43) and 26% (7/27) also decreased in abundance in the *F. oxysporum* f. sp. *lini* alone inoculation at 9 dpi or 14 dpi, respectively. However, of the transcripts that decreased in abundance following the combined inoculation, 40% (17/43; 9 dpi) and 70% (20/27; 14 dpi) also decreased in abundance following the inoculation by *R. irregulare* alone.

Another interesting comparison that might help to elucidate the bio-protective effects of *R. irregulare* were the genes up-regulated at 9 dpi in both the combined treatment and the *R. irregulare* treatment. Most of these genes were related to AM symbiosis and were not differentially expressed following *F. oxysporum* f. sp. *lini* treatments. For instance, nitrate transporter 2:1 (Lus10016121), and putative cell wall modification-related genes (Lus10033360, Lus10039881, Lus10008213). Finally, two peroxidases (Lus10025256 and Lus10001228), a cytochrome P450,79 B 2 (Lus10031145) and a LTP/seed storage 2S albumin superfamily (Lus10022066) that were not induced by *F. oxysporum* f. sp. *lini* alone were induced in both flax- *R. irregulare* and the combined treatment.

## 4 Discussion

**Figure 8:**
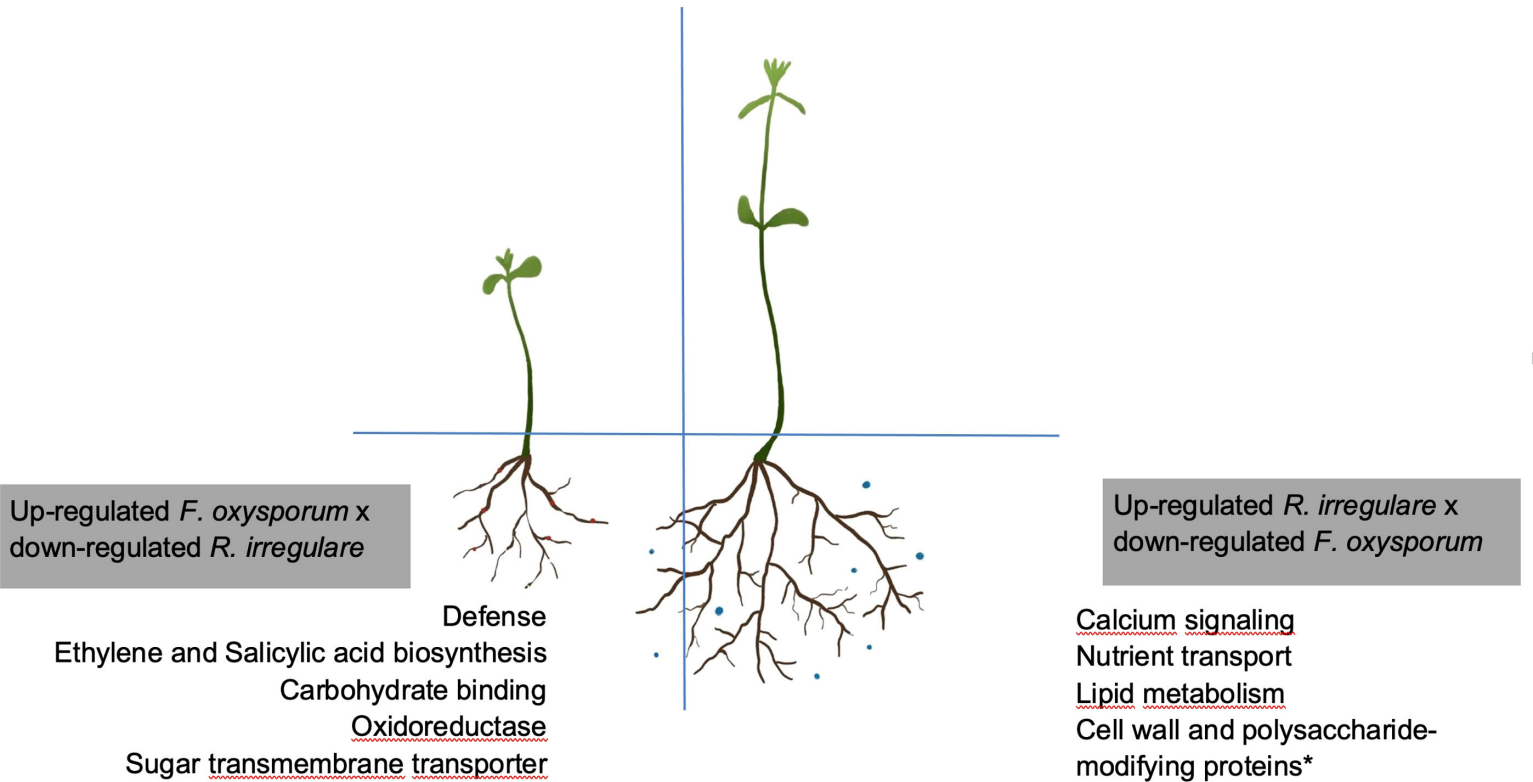
Schematic representation of opposite modifications in the flax transcriptome upon infection with *F. oxysporum* f. sp. *lini* or symbiosis with *R. irregulare*. Boxes shown genes that were exclusively up-regulated by *F. oxysporum* f. sp. *lini* alone or *R. irregulare alone*, when these two treatments were compared. *indicates that other genes involved with cell wall modifications, not represented here, might be up-regulated by *F. oxysporum* f. sp. *lini* ^©^ Sara Gagnon. This work is a derivative from (Fiorilli et al., 2018), used under a Creative Commons Attribution 4.0 International License (http://creativecommons.org/licenses/by/4.0/).

In our study we used the mutualist, *R. irregulare*, and the pathogen, *F. oxysporum* f. sp. *lini*, as models to investigate the molecular mechanisms deployed at the initial interactions between flax and AMF that contribute to minimize the effects of pathogenic fungi. We note that the early recognition of partners during symbiotic interactions is the foundation for establishing a successful relationship, as well as activating plant defense mechanisms (Bedini et al., 2018; García-Garrido and Ocampo, 2002 Unraveling the mechanisms by which mutualistic fungi establish healthy relationships with plants by navigating the plant immune system, despite sharing invasion strategies with pathogens, remains challenging (Chen et al., 2022). Employing a multi-omics approach presents an avenue for dissecting these complex relationships (Chen et al., 2022). Most of our understanding of AMF bio-protective effects to pathogenic fungi comes from plants colonized by mycorrhizal fungi, overlooking the effects of early interactions (Akköprü and Demir, 2005; Fiorilli et al., 2011, Fiorilli et al., 2018). Exploring the roots, the starting point for many plant-fungi interactions, and examining the transcriptome dynamics during colonization and infection, is essential to understand these processes and develop effective strategies for combating fungal diseases (Rauyaree et al., 2001). Our research unveiled two key mechanisms for symbiosis: the regulation of genes directly associated with AMF development and genes directly associated with defense responses (**Figure 8**).

### 4.1 Mycorrhization establishment versus pathogenesis

Our research suggests that the plant response to AMF prioritizes expression of genes directly involved in mutualism rather than defense (**Table 6**), while the opposite behavior was observed with the pathogenic fungus (**Table 7**). This is consistent with previous reports that AMF treatment does not induce major defense mechanisms in plants (Smith and Read, 2008).

In our study, genes related to nutrient uptake and cell wall modifications were up-regulated by the *R. irregulare-*alone treatment (**Table 6**). *R. irregulare* also seemed to manipulate defense responses in flax by affecting gene expression directly in the combined treatment (**Table 6**). Several transcripts showed decreased abundance in the combined treatment compared to *F. oxysporum* f. sp. *lini* alone, and these transcripts similarly decreased following *R. irregulare* alone treatment (**Figure 6**). Genes induced for symbiosis by *R. irregulare* alone continued to be induced in the combined treatment, while most genes involved in the defense response induced by *F. oxysporum* f. sp. *lini* alone had decreased transcript abundance in the combined treatment (**Tables 6** and **7**). This suggests that *R. irregulare* directly coordinates the regulation of defense genes in response to the pathogen, supporting the notion of extensive transcriptional reprogramming during AMF colonization (Smith and Read, 2008). Previous research indicated a suppression of defense mechanisms in plant roots by AMF to favor colonization, further emphasizing the intricate interplay between mycorrhization and defense responses (Vangelisti et al., 2018).

While both of the fungi tested induced responses in plants, the number of DEGs was notably higher in response to *F. oxysporum* f. sp. *lini* compared to *R. irregulare* (**Figure 2**). This discrepancy could be related to a more aggressive growth rate of the pathogenic fungus, or could be a rapid plant response to stress, contrasting with a potentially more passive response to a mutualist.

#### 4.1.1 Mutualism

The main categories of genes related to mutualism and up-regulated by *R. irregulare* were: nutrient uptake and transporters; and cell wall modifications. The majority of genes up-regulated in flax in the presence of *R. irregulare* occurred at 14 dpi, when compared with 9 dpi (**Figure 6**). We will further discuss some of the genes involved in the pre-colonization events leading to establishment of mutualism.

##### 4.1.1.1 Nutrient uptake and transporters

In our study, nutrient transporter genes were up-regulated in flax under *R. irregulare* treatment (Lus10010696, Lus10030872, and Lus10016121; **Table 6**). Previous studies have identified nitrate, phosphate and ammonium transporters in AMF symbiosis (Benedito et al., 2010; Fiorilli et al., 2018; Guether et al., 2009). These transporters play essential roles in plant nutrition, metabolism and signaling, affecting plant growth, development and environmental responses (Benedito et al., 2010), therefore promoting mutualistic interactions.

*R. irregulare* induced a nodulin transporter (Lus10010696; **Table 6**) in both single and combined treatments, whereas it was repressed by *F. oxysporum* f. sp. *lini*. Notably, nodulin transporters, known genes of rhizobial symbiosis pathways, also played a role in early AMF symbiosis, even before the physical interaction between the plant and fungi (Kosuta et al., 2003).

The nitrate (NO_3_^-^) transporter (Lus10016121) was up-regulated by *R. irregulare* in both the individual and combined treatment, but repressed by the *F. oxysporum* f. sp. *lini-*alone treatment in our study (**Table 6**). This transporter plays a role in plant nutrition and lateral root development, therefore promoting symbiosis. The potential increased nitrogen uptake in the combined treatment could contribute to larger and higher biomass plants than those inoculated with *F. oxysporum* f. sp. *lini a*lone (**Figure 1**, **2**). Nitrogen (N) is also an important key signal for pathogenesis, which might be induced in nitrogen-limiting conditions *in vitro* (Snoeijers et al., 2000). Besides, the expression of virulence genes in *F. oxysporum* f. sp. *lini* can be controlled by the nitrogen sources (López-Berges et al., 2010). For example, the presence of ammonium represses the expression of *F. oxysporum* f. sp. *lini* genes that control vegetative growth and root adhesion, therefore reducing infection (López-Berges et al., 2010). High N levels have been associated with reduced severity of infections caused by facultative parasites like *Fusarium* (Dordas, 2009). Our findings suggest that *R. irregulare*, by enhancing N uptake in flax, might mitigate *F. oxysporum* f. sp. *lini* virulence, resulting in a less pronounced activation of defense genes (**Table 5**) compared with the robust response induced by the *F. oxysporum* f. sp. *lini* individual treatment.

##### 4.1.1.2 Cell wall modifications

Cell wall modification-related genes were differentially expressed across all treatments. *R. irregulare* induced the expression of many genes related to cell wall modifications, in both alone and combined treatments, while *F. oxysporum* f. sp. *lini* alone repressed the same genes at 14 dpi. These included an extensin (Lus10008213, pollen Ole e 1 allergen, extensin family) and two hydroxyproline-rich glycoproteins (HRGPs) (Lus10033360, Lus10039881) (**Table 6**).

Other research has highlighted the up-regulation of genes involved in cell wall metabolism by mycorrhizal fungi, such as HRGPs (Wu et al., 2017). HRGPs strengthen cell wall structures, restricting fungal spread in plants (Basavaraju et al., 2009; Ribeiro et al., 2006; Wu et al., 2017). The deployment of these local responses, such as cell wall modifications, by AMF in flax roots suggests a strategy to impede disease progression. Additionally, peroxidases, implicated in cell wall cross-linking (Basavaraju et al., 2009) were up-regulated by *R. irregulare* and down-regulated by *F. oxysporum* f. sp. *lini* in our study (Lus10025256, Lus10006534, Lus10001228; **Table 6**). This evidence shows the importance of peroxidases to defense mechanisms, and the pathogen strategy to evade the plant immune system by negatively interfering with the expression of defense genes.

#### 4.1.2 Defense responses

Among the mechanisms involved in the regulation of defense responses are: direct regulation of proteins (such as PRs and chitinases, **Table 7**) and secondary metabolites involved in plant defenses (**Table 6**, **7**), hormone regulation and systemic acquired resistance (**Table 5**).

There was a reduction in the number of genes related to defense [(GO:0006952); **Table 5**, **7**)] and immune responses [(GO:0006955); **Table 5**, **7**)] in the combined treatment, when compared to the *F. oxysporum* f. sp. *lini*-alone treatment. Interestingly, genes belonging to these two categories, defense and immune response [GO:0006952 and GO:0006955; **Table 5**, **7**)], were down-regulated by *R. irregulare* both at 9 and 14 dpi. This means that the addition of *R. irregulare* in the combined treatment, resulted in a constriction of flax transcriptomic defense responses when compared to the *F. oxysporum* f. sp. *lini*-alone treatment.

It has been shown that when AM symbiosis triggers defense responses, these are deployed for a short time and later repressed (Smith and Read, 2008). Moreover, the extent to which these responses are activated is much less than the defenses deployed upon the pathogen attack (Smith and Read, 2008). These studies support the idea that AMF can modulate defense responses in plants. However, in our studies, we observed a great suppression of defense responses in the early stages of AMF symbiosis. This observation may be explained by the fact that we are studying the events preceding observable colonization and defense-related mRNAs increase later in colonization when cells contain arbuscules (Smith and Read, 2008). Another possibility, as discussed before, is that the changes in flax transcriptome induced by AMF (such as cell wall modifications and N uptake) inhibit or delay *F. oxysporum* f. sp. *lini* growth and spread, reflecting a reduction of the number of DEG in comparison with *F. oxysporum* f. sp. *lini* treatment. It is also possible that AMF directly suppresses many defense responses to favor colonization (Guether et al., 2009; Vangelisti et al., 2018). In fact, it seems that both mechanisms are taking place simultaneously in our study.

It is noteworthy that despite *R. irregulare* attenuating the expression of most defense genes in the combined treatment, plants did not show any signs of disease (**Supplementary Figure 1**). We speculate that besides the beneficial responses triggered by AMF beneficial relationships (such as nutrient uptake, hormone regulation) the defense genes that were induced in the combined treatment were sufficient to suppress the disease. For instance, PR genes (Lus10004410 and Lus10012479), and one chitinase (Lus10003231) were strongly induced in the combined treatment (log_2_ fold change > 4) and *F. oxysporum* f. sp. *lini-*alone treatment (**Table 7**). Our observations are consistent with the idea that AMF does not activate major defense responses in plants, rather AMF induces slight changes in plants defense gene expression that are later suppressed but are sufficient to promote immunity (Smith and Read, 2008).

It is interesting to note that among the defense genes suppressed by *R. irregulare* in the combined treatment, a few were up-regulated in flax in response to *F. oxysporum* f. sp. *lini* only at 18 dpi in previous studies (Galindo-Gonzalez and Deyholos, 2016): for instance, several Kunitz, aspartyl proteases, and ethylene responsive factors. This result suggests that these genes may be related to disease progress in flax in response to *F. oxysporum* f. sp. *lini*.

##### 4.1.2.1 Hormone signaling

Both types of fungi altered the biosynthesis of hormones in plants although often in contrasting ways. For instance, JA biosynthesis, and SA-responsive pathogenesis-related genes were mainly increased in the flax transcriptome by *F. oxysporum* f. sp. *lini*-alone treatment (Enriched GO categories: jasmonic acid (JA) metabolic processes (GO:0009694), and systemic acquired resistance (GO:0009627); **Table 5**) whereas ethylene-responsive genes were mainly down-regulated by the *R. irregulare*-alone treatment (**Supplementary Tables S4**, **S6**). However, JA biosynthesis genes were up-regulated in both treatments.

Hormone influence on plant-fungal interactions varies according to the type of fungus, location of interaction and stage of colonization. For instance, hormone signaling during AMF colonization and establishment in plants is highly diverse and involves an extensive signaling network (Bucher et al., 2014). It has been suggested that ethylene and SA limit AMF entry in plants, and therefore negatively affect AMF colonization (Mukherjee and Ané, 2011). Moreover, studies with mutant plants have shown that JA deficiency reduces mycorrhization (Bucher et al., 2014). Our results suggest that AMF decreases ET production (e.g. Lus10011829, Lus10004368, **Supplementary Table S6**) and genes related to SA (e.g. Lus10041460, and Lus10020686, **Table 7**) but increases JA (Lus10027648, Lus10039911.g; **Supplementary Table S2**) to favor colonization. Although progress has been made in characterizing the molecular mechanisms associated with AMF pre-symbiotic signaling, it is still not clear if the role of these phytohormones is essential for AMF symbiosis (Bucher et al., 2014). More studies are necessary to elucidate many gaps in the knowledge of AMF pre-symbiotic signaling. In contrast, *F. oxysporum* f. sp. *lini* induces the expression of genes related to ET, SA and JA, which are involved in many signaling defense pathways and have been associated with defense to *F. oxysporum* f. sp. *lini* in flax transcriptomic studies (Galindo-González & Deyholos, 2016).

##### 4.1.2.2 Secondary metabolism

An important gene possibly involved in defense responses that was up-regulated in the *R. irregulare* alone treatment and combined treatment, and that was down-regulated by *F. oxysporum* f. sp. *lini* was cytochrome 79 B2 (CYP79B2) (Lus10031145; **Table 6**). In *Arabidopsis*, CYP79B2 is involved in the biosynthesis of precursors of indole-3-acetic acid (IAA), and indole glucosinolates and camalexin (Bak et al., 2011). Conversely, other members of the cytochrome P450 (CYP) family were up-regulated by *F. oxysporum* f. sp. *lini* alone treatment at 9 and 14 dpi (Lus10014284, cytochrome BC1 synthesis; Lus10030189, cytochrome P450 71B10; Lus10013150, cytochrome P450 82C; and Lus10038200, Lus10011658, Lus10025901, Lus10025902, cytochrome P450 82C4; **Table 7**). The CYP transcripts up-regulated by *F. oxysporum* f. sp. *lini* in the single treatment were mostly down-regulated by *R. irregulare* single treatment and had mixed patterns in the combined treatment in our studies (**Table 7**). The up-regulation of several CYP genes in response to *F. oxysporum* f. sp. *lini* in flax was observed in previous studies, supporting our findings (Galindo-Gonzalez and Deyholos, 2016).

Other genes likely involved with secondary metabolite production include an O-methyltransferase (OMT, Lus10006691; **Table 7**), and geranylgeranyl pyrophosphate synthase (GGPPS, Lus10023259, Lus10017624; **Supplementary Table S7**). Both of these were up-regulated by *F. oxysporum* f. sp. *lini* in the single and combined treatments. OMT genes might be involved in the methylation of secondary metabolites that play important roles in the synthesis of antimicrobial compounds (phytoalexins) and lignin biosynthesis (Lam et al., 2007). GGPP is a key isoprenoid (Coman et al., 2014). A member of 2-oxoglutarate (2OG) and Fe(II)-dependent oxygenase protein (2-ODDs) was up-regulated by *F. oxysporum* f. sp. *lini* in single and combined treatments at 14 dpi. These gene products are considered one of the most versatile types of oxidative enzymes, participating in a wide range of reactions of metabolism, for example hormones and secondary metabolites involved in plant defenses (Farrow and Facchini, 2014). These results are supported by the work of Galindo-Gonzalez and Deyholos (2016), who found that several of these processes/genes were enriched/up-regulated in flax (whole plant) in response to *F. oxysporum* f. sp. *lini*.

##### 4.1.2.3 Calcium signaling

Calcium signaling is important for pathogen defense. For instance, different transcripts involving calcium signaling (e.g. Lus10027261, Lus10028657, Lus10034463, Lus10026291, Calcium-binding EF-hand family protein; Lus10006523, Calcium-dependent lipid-binding (CaLB domain) family protein; **Supplementary Table S4**) were up-regulated only by the *F. oxysporum* f. sp. *lini* alone treatment at 9 and 14 dpi.

We also observed an up-regulation of MAPK (Lus10025986, Lus10001081.g; **Supplementary Table S4**) by *F. oxysporum* f. sp. *lini* -alone treatment at 9 and 14 dpi in flax. Similar results were found by Galindo-Gonzalez and Deyholos (2016) when analyzing flax transcriptome responses to *F. oxysporum* f. sp. *lini* and were associated with the signal transduction cascade, activated in response to pathogen perception, that would culminate with the further activation of several defense responses, like biosynthesis of hormones, PR proteins, polyamines, isoprenoids, ROS and others (Galindo-Gonzalez and Deyholos, 2016).

Calcium signaling is also required for AMF symbiosis and it is the first change that AMF elicits in plants in pre-symbiotic events through Myc factors (Bonfante and Genre, 2010). The rapid and transient calcium spiking triggers a signaling pathway known as the common symbiosis pathway (Parniske, 2008). In our studies a Ca-dependent lipid-binding family (Lus10039259, C2 domains) was activated at 9 dpi by inoculation with *R. irregulare* in single and combined treatments (**Table 6**). Other transcriptome studies involving AMF symbiosis have found the up-regulation of genes encoding calcium-depending protein kinases (Vangelisti et al., 2018).

The dual involvement of calcium signaling in both pathogen defense and AMF symbiosis highlights its intricate role in orchestrating plant responses to diverse environmental challenges.

### 4.2 Bio-protective effects of *R. irregulare* in flax roots

We believe that the bioprotective effect observed (**Figure 1**) is due to multiple mechanisms. For instance, despite the slight changes in genes related to nutrient uptake, where only one nitrate transporter was up-regulated in the combined treatment (**Table 6**), flax showed increased shoot length (**Figure 1A**) and biomass compared to either *F. oxysporum* f. sp. *lini* or AMF alone (**Figure 1B**). These data are consistent with the concept that AMF symbiosis promotes positive growth responses due to increase in the availability of growing-limiting nutrients (Smith and Read, 2008). These changes also occur to the *R. irregulare* alone treatment and are supported by the work of Fiorilli et al. (2018).

Our work also showed that co-inoculation with *R. irregulare* and *F. oxysporum* f. sp. *lini* provoked changes in phytohormone-related genes, which may also influence flax growth and defense responses. AMF preferentially triggered genes involved in the JA pathway (Lus10027648, Lus10039911; **Supplementary Table S2**), probably leading to MIR (Cameron et al., 2013), whereas *F. oxysporum* f. sp. *lini* preferentially triggered SA (e.g. Lus10041460, and Lus10020686, **Table 7**) and JA pathway (Lus10027648, Lus10039911.g; **Supplementary Table S2**), leading to SAR-like resistance. However, genes involved in the JA pathway were not strongly induced in the combined treatment (FC > 0 <1; **Supplementary Table S2**), for reasons that cannot currently be explained. Modifications in the cell wall possibly interfered with pathogenic colonization and spread, as suggested by the literature (Basavaraju et al., 2009; Ribeiro et al., 2006; Wu et al., 2017). Even though there was a general repression of defense genes to counter the pathogen attack, some defense genes were continuously up-regulated, such as PR (Lus10015339, Lus10020493, and Lus10012479), spermidine (Lus10005358; **Table 6**, 7), chitinase (Lus10003231, Lus10010866, and Lus10024366) and peroxidase (Lus10012684) (**Table 7**). These data suggest that the activation of the host immune system was triggered mainly in response to the pathogenic fungi, but possibly modulated by AMF. It has been shown that the effects of AMF on defense genes, such as chitinases and PRs proteins (which were up-regulated in our studies in the *F. oxysporum* f. sp. *lini* single and combined treatments) enhance local resistance to pathogen penetration in plants, and as well have long-distance effects, respectively (Ismail and Hijri, 2012).

In summary, high throughput RNA-Seq data revealed that AMF mitigates the reductions in shoot length and weight in flax phenotype caused by the pathogen, meaning that AMF induces bio-protective effects in flax. Several of the genes reprogrammed have been described in mycorrhizal plants, however, our results showed remarkable effects at a pre-symbiotic stage. Defense responses were mainly suppressed by *R. irregulare* in the single and combined treatment, suggesting that AMF affects defense genes directly and indirectly.

Finally, in our study it was clear that the bio-protective effects of *R. irregulare* were associated with a balance between enhancing plant nutritional status, and morphological and physiological changes in response to AMF symbiosis development and as well, regulation of defense responses. The changes promoted by AMF on the flax transcriptome were not sufficient to completely inhibit the growth of the pathogen, since *F. oxysporum* f. sp. *lini* was re-isolated from flax roots in the combined treatment. Therefore, the activation of plant defenses against *F. oxysporum* f. sp. *lini*, even though they were reduced, were also essential for the positive effects on flax growth observed in the combined treatment in comparison to flax phenotype when inoculated with *F. oxysporum* alone f. sp. *lini* (**Figures 1**, **2**, **5**).

Overall, we hypothesize that the effects observed on flax DEGs triggered by *R. irregulare*, directly and indirectly, limited *F. oxysporum* f. sp. *lini* growth, and consequently, restricted the transcriptional response to *F. oxysporum* f. sp. *lini* in the flax-combined treatment. Although we were not able to compare the amount of *F. oxysporum* f. sp. *lini* that colonized the roots in the single and combined treatment, the healthy phenotype observed in the combined treatment despite the presence of *F. oxysporum* f. sp. *lini*, suggests that the progress of disease was at least delayed. To develop a full picture of the effects of *R. irregulare* on *F. oxysporum* f. sp. *lini* growth and spread in flax, additional studies will be needed to measure the amount of these fungi in flax roots.

The genes presented in this work could be tested for single and co-expression in engineered plants, with the aim of phytopathogen protection. Plant crop engineering often focuses on the manipulation of obvious genes involved in disease resistance, using strategies such as stacking R genes, over-expression of PR proteins and other antimicrobial peptides, augment of plant immune receptors, among others. We suggest that further studies should consider combining different strategies for enhancing plants tolerance to pathogens, and the genes that we found in this study would be interesting targets. For instance, tomato plants transformed with a binary vector co-expressed a snakin and an extensin-like protein, and presented enhanced tolerance to *Clavibacter michiganensis* (Balaji and Smart, 2012). A delay in symptom development and a reduction of canker lesions were observed relative to non-transgenic plants (Balaji and Smart, 2012).

## 5 Conclusion

To conclude, flax RNA-Seq changes upon inoculation with pathogenic or mutualist fungi were performed with success. We believe that the main mechanisms underlying mycorrhization before colonization are the nutrient uptake and cell wall modifications, followed by hormone regulation and secondary metabolite production. In contrast, upon a pathogen attack, flax plants respond mainly by activating defense genes, which despite an attempt at protection, is also reflected in stalling flax growth in our studies. Finally, when co-inoculated, both fungi interfered with plant responses, where a combination of mechanisms related to AMF symbiosis development and defense mechanisms were maintained from each alone. *R. irregulare* seems to interfere with the *F. oxysporum* f. sp. *lini* infection, where a great reduction in the number of genes in the *F. oxysporum* f. sp. *lini* alone treatment was observed. Our findings suggest a great regulation of flax defense by *R. irregulare*. These data will aid in understanding the early relationships of mutualism and pathogenicity in plants, as well as in the selection of target genes for genetic improvement.

## Data Availability

The datasets presented in this study can be found in online repositories. The names of the repository/repositories and accession number(s) can be found below: https://www.ncbi.nlm.nih.gov/, PRJNA432224.

## Supplementary material

Supplementary Table 1 List of GO AgriGO gene enrichment categories Data.

Supplementary Table 2 9 dpi *R. irregulare* inoculum vs. control, list of significantly differentially expressed genes (DGE).

Supplementary Table 3 14 dpi *R. irregulare* inoculum vs. control, list of significantly differentially expressed genes (DGE).

Supplementary Table 4 9 dpi *F.oxysporum* f. sp. *lini* inoculum vs. control, list of significantly differentially expressed genes (DGE).

Supplementary Table 5 14 dpi *F.oxysporum* f. sp. *lini* inoculum vs. control, list of significantly differentially expressed genes (DGE).

Supplementary Table 6 9 dpi combined inocula vs. control, list of differentially significantly expressed genes (DGE).

Supplementary Table 7 14dpi combined inocula vs. control, list of significantly differentially expressed genes (DGE).

Supplementary Figure 1 Showing effects of inocula on seedlings.
