# Supplementary material for: Gene expression signatures of mutualism and pathogenesis in flax roots
Source: Front Plant Sci. 2024 Oct 10;15:1415082. doi: 10.3389/fpls.2024.1415082 (PMC11499196; doi:10.3389/fpls.2024.1415082)
Supplement: Supplementary Figure 1 — Showing effects of inocula on seedlings. [file DataSheet8.pdf]

Supplemental Image S1

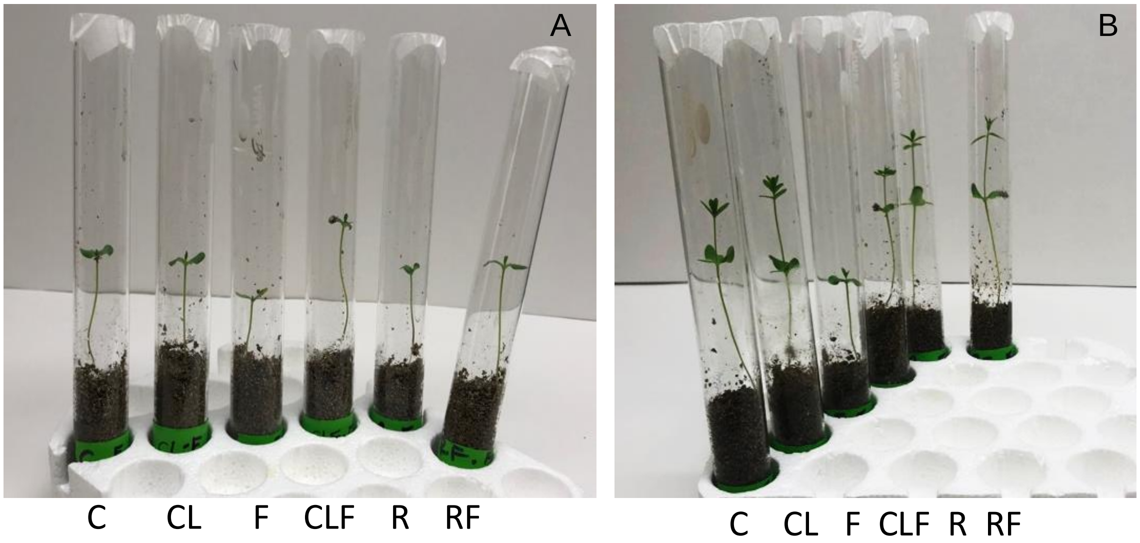

Disease symptoms 9 days post infection (A) and 14 days post infection (B).
Treatments are: C, uninoculated control; Cl, *Clonostachys rosea*; F, *Fusarium oxysporum*; ClF, CI *Clonostachys rosea* + *Fusarium oxysporum*; R, *Rhizoglomus irregulare*; RF, *Rhizoglomus irregulare* + *Fusarium oxysporum. Clonostachys rosea* is not discussed as part of the experiments presented here.
